# Supplementary material for: Functional screening in human HSPCs identifies optimized protein-based enhancers of Homology Directed Repair
Source: Nat Commun. 2024 Mar 23;15:2625. doi: 10.1038/s41467-024-46816-5 (PMC10960832; doi:10.1038/s41467-024-46816-5)
Supplement: Supplementary file 1 — Supplementary Information [file 41467_2024_46816_MOESM1_ESM.pdf]

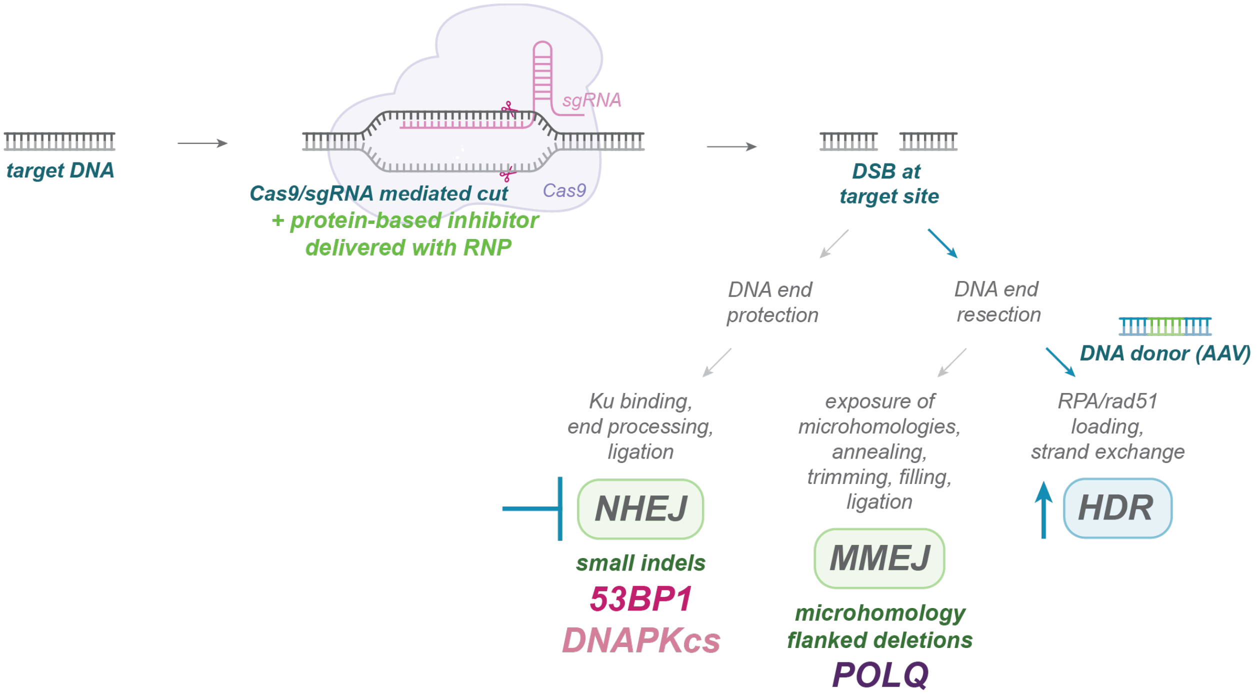

***Supplementary Figure 1.1*: Boosting HDR-based gene editing outcomes through protein-based inhibitors of key DNA repair enzymes.** Schematic outlining the impact antagonists of key DNA repair enzymes can have on the various editing outcomes that occur after a Cas9-mediated site-specific double strand break (DSB). Key target enzymes for NHEJ and MMEJ pathways are listed below. Inhibition of 53BP1 or DNAPKcs is predicted to increase HDR, as shown in blue arrows.

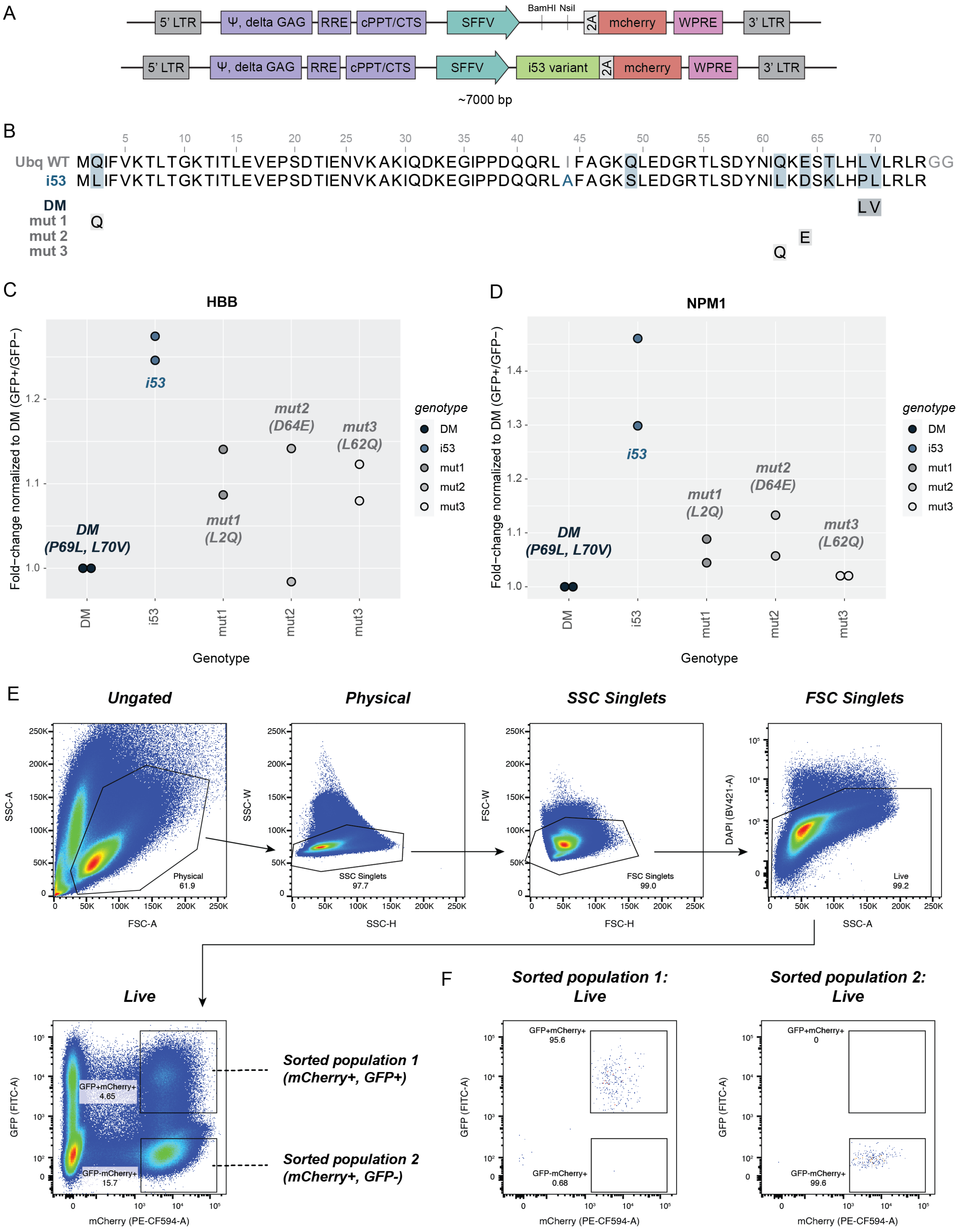
A
5' LTR
Ψ, delta GAG
RRE
cPPT/CTS
SFFV
BamHI NsiI
2A
mcherry
WPRE
3' LTR
i53 variant
~7000 bp
B
Ubq WT MQIFVKTLTGKTITLEVEPSDTIENVKAKIQDKEGIPPDQQRLIFAGKQLEDGRTLSDYNIQKESTLHLVLRLRGG
i53 MLIFVKTLTGKTITLEVEPSDTIENVKAKIQDKEGIPPDQQRLAFAGKSLEDGRTLSDYNILKDSKLHPLLRLR
DM
mut 1
mut 2
mut 3
C
HBB
D
NPM1
Fold−change normalized to DM (GFP+/GFP−)
Genotype
genotype
DM
i53
mut1
mut2
mut3
DM (P69L, L70V)
mut1 (L2Q)
mut2 (D64E)
mut3 (L62Q)
E
Ungated
Physical
SSC Singlets
FSC Singlets
Live
FSC-A
SSC-A
SSC-H
SSC-W
FSC-H
FSC-W
DAPI (BV421-A)
Physical 61.9
SSC Singlets 97.7
FSC Singlets 99.0
Live 99.2
GFP (FITC-A)
mCherry (PE-CF594-A)
GFP+mCherry+ 4.65
GFP-mCherry+ 15.7
Sorted population 1 (mCherry+, GFP+)
Sorted population 2 (mCherry+, GFP-)
F
Sorted population 1: Live
Sorted population 2: Live
GFP+mCherry+ 95.6
GFP-mCherry+ 0.68
GFP+mCherry+ 0
GFP-mCherry+ 99.6

***Supplementary Figure 1.2*: Lentiviral pooled screening design and validation.** (A) A lentiviral transfer plasmid was built with restriction sites upstream of a T2A-mCherry-WPRE cassette to easily clone in sequences of interest. Placement of a T2A-mCherry tag at the 3' end of the protein variants allowed for independent expression of both the protein variant and mCherry post translation, enabling fluorescence-based monitoring of cells expressing protein variants of interest. (B) To validate the lentiviral-based screening system in HSPCs, different variants of i53 were cloned into the lentiviral vector: i53 (positive control), a previously reported dead variant of i53 (DM, negative control), and three variants of i53 that have been previously reported to have decreased (but detectable) binding to the 53BP1 Tudor domain relative to i53 ("mut1" = L2Q, "mut2" = D64E, and "mut3" = L62Q). Plasmids encoding these five i53 variants were pooled together to generate a mock library. CD34+ HSPC cells were transduced using lentivirus packaged with the mock library and edited in duplicate at the *HBB* locus using HBB-UbC-GFP AAV6 (MOI = 2500) and *NPM1* locus using NPM1-GFP AAV6 (MOI = 2500). NGS analysis of the gDNA purified from sorted mCherry+GFP+ and mCherry+GFP- populations indicated differential variant enrichment in the populations relative to the DM control for editing at (C) *HBB* and (D) *NPM1*. (E) Representative flow cytometry plots showing the gating strategy used to sort edited GFP positive cells and GFP negative cells in transduced (mCherry+) HSPC populations. Source data are provided as a Source Data file. (F) Purity of sorted populations was confirmed by post-sort purity checks.

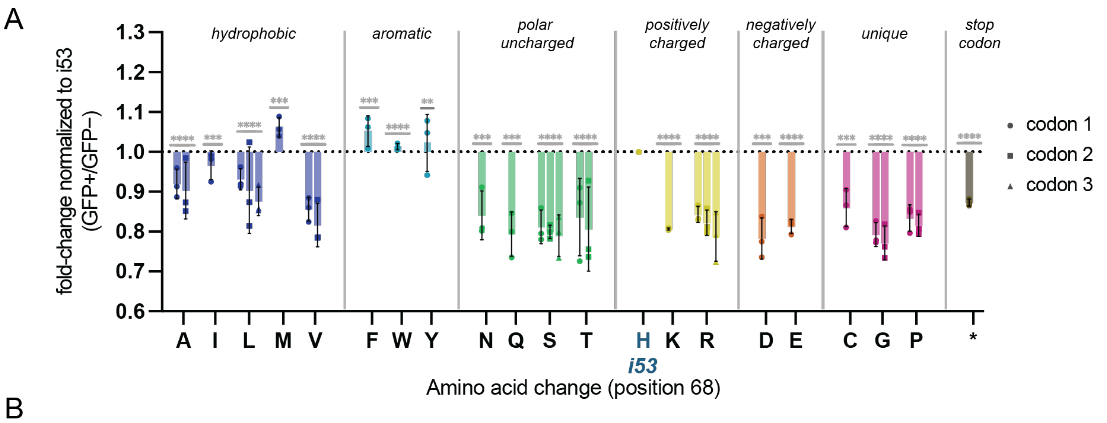

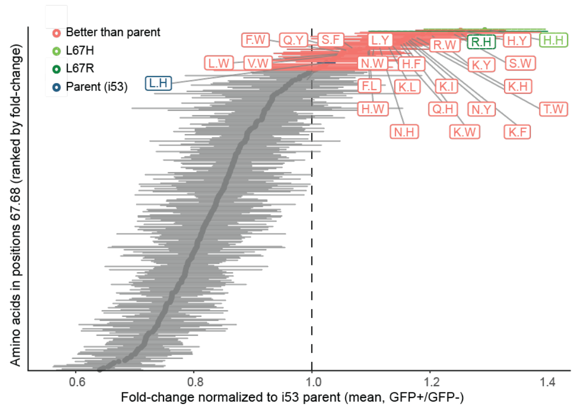

rank	aa1.aa2	fcmean
1	H.H	1.29
2	H.Y	1.25
3	R.H	1.24
4	S.W	1.23
5	K.Y	1.20
6	R.W	1.19
7	K.H	1.17
8	T.W	1.16
9	K.F	1.16
10	N.Y	1.16
11	K.I	1.15
12	K.W	1.15
13	H.F	1.15
14	Q.H	1.13
15	K.L	1.13
16	N.H	1.11
17	N.W	1.11
18	H.W	1.10
19	F.L	1.09
20	L.Y	1.08
21	S.F	1.07
22	Q.Y	1.07
23	F.W	1.05
24	L.W	1.05
25	V.W	1.05

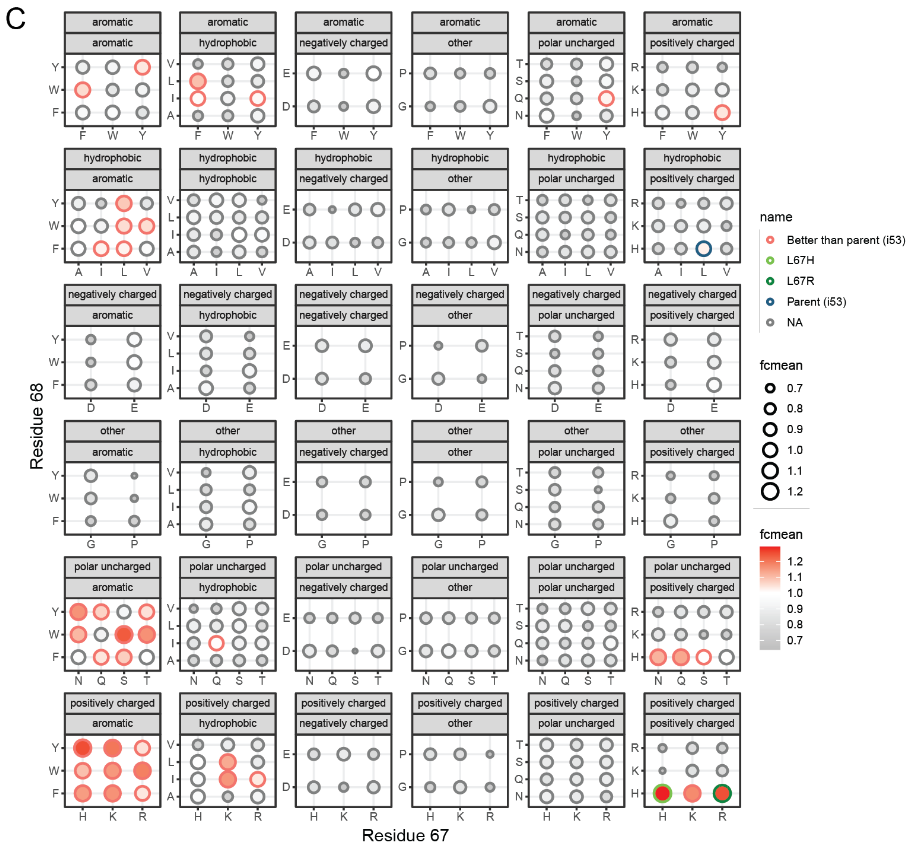

***Supplementary Figure 1.3* (related to Figure 1C): Screening libraries targeting i53 residues 67 and 68**. (A) A focused saturation mutagenesis library was generated using NNK primers to independently vary the amino acid identity of residue 68 of i53 (library size = 32 codon encoding for 20 amino acids). Differential enrichment of variants containing mutations at residue 68 in the mCherry+GFP+ population was calculated from the variant abundance in sorted mCherry+GFP+ and mCherry+GFP- populations relative to i53 (H68). CD34+ HSPC cells were transduced using lentivirus packaged with this focused NNK library and edited at the *HBB* locus using HBB-UbC-GFP AAV6 (MOI = 1250). *n 3* separate pooled analyses and mean ± SD depicted. Each bar represents a unique codon for that amino acid. Of the 19 new variants tested in this library, three were found to be significantly enriched relative to parent WT i53 (H68). ***p < 0.01; ***p-< 0.001' ****p < 0.0001. T*wo-tailed t-test with Holm-Šídák correction for multiple comparisons. (B) A combinatorial library was designed to explore all amino acid combinations (excluding cysteine and methionine, library size = 324 variants) at i53 parent sequence L67 and H68 at the 53BP1/i53 binding interface. CD34+ HSPC cells were transduced using lentivirus packaged with the combinatorial library and edited in triplicate at the *HBB* locus using HBB-UbC-GFP AAV6 (MOI = 2500). Differential variant enrichment was calculated from the variant abundance in sorted mCherry+GFP+ over mCherry+GFP- and ranked relative to parent i53, shown in blue. Hits L67R and L67H from the NNK screen are shown in green, respectively. Variants with average fold change over parent (i53) larger than 1.0 are highlighted in red (25 variants, or 7.7%). (C) Dot plot representation of variant fold change enrichment as in (B), clustered by amino acid properties (amino acid variations of residues 67 and 68 shown on the x-axis and y-axis, respectively). Note amino acid properties listed for each cluster shown refer to those for residue 67 above and to residue 68 below. *n = 3* separate pooled libraries. (A-C) Source data are provided as a Source Data file.

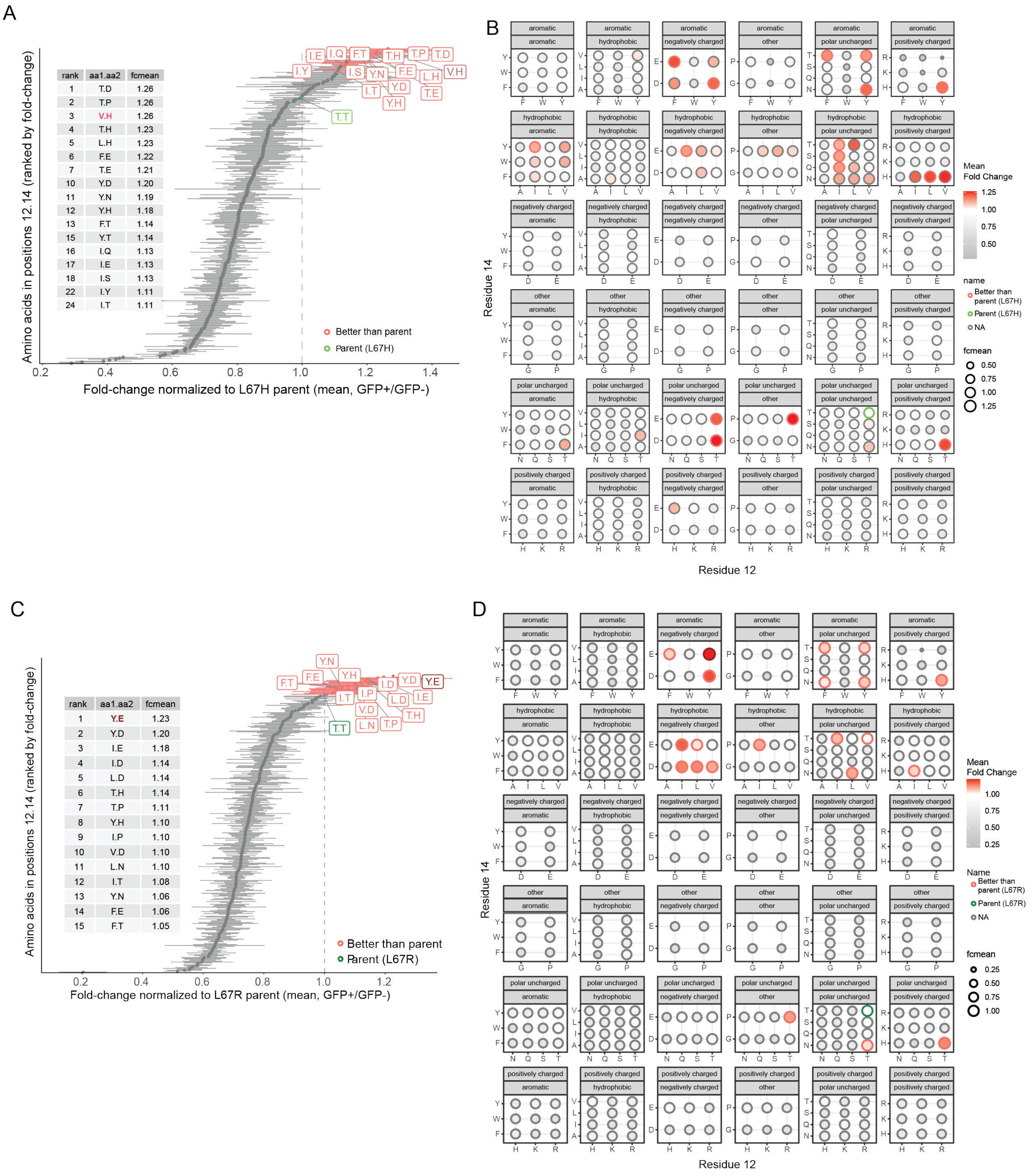
A
Amino acids in positions 12.14 (ranked by fold-change)
Fold-change normalized to L67H parent (mean, GFP+/GFP-)
rank aa1.aa2 fcmean
1 T.D 1.26
2 T.P 1.26
3 V.H 1.26
4 T.H 1.23
5 L.H 1.23
6 F.E 1.22
7 T.E 1.21
10 Y.D 1.20
11 Y.N 1.19
12 Y.H 1.18
13 F.T 1.14
15 Y.T 1.14
16 I.Q 1.13
17 I.E 1.13
18 I.S 1.13
22 I.Y 1.11
24 I.T 1.11
Better than parent
Parent (L67H)
B
Residue 14
Residue 12
Mean Fold Change
name
Better than parent (L67H)
Parent (L67H)
NA
fcmean
C
Amino acids in positions 12.14 (ranked by fold-change)
Fold-change normalized to L67R parent (mean, GFP+/GFP-)
rank aa1.aa2 fcmean
1 Y.E 1.23
2 Y.D 1.20
3 I.E 1.18
4 I.D 1.14
5 L.D 1.14
6 T.H 1.14
7 T.P 1.11
8 Y.H 1.10
9 I.P 1.10
10 V.D 1.10
11 L.N 1.10
12 I.T 1.08
13 Y.N 1.06
14 F.E 1.06
15 F.T 1.05
Better than parent
Parent (L67R)
D
Residue 14
Residue 12
Mean Fold Change
Name
Better than parent (L67R)
Parent (L67R)
NA
fcmean

Supplementary Figure 1.4 **(related to Figure 1D): Screening of a combinatorial library targeting residues 12 and 14 of L67H and L67R. (A)** A combinatorial library was designed to explore all amino acid combinations (excluding cysteine and methionine, library size = 324 variants) at additional residues (T12, T14) at the 53BP1/i53 interface using L67H as the parent sequence. CD34+ HSPC cells were transduced using lentivirus packaged with the combinatorial library and edited in triplicate at the *HBB* locus using HBB-UbC-GFP AAV6 (MOI = 2500). Differential variant enrichment was calculated from the variant abundance in sorted mCherry+GFP+ over mCherry+GFP- populations and graphed relative to parent L67H, shown in green. Variants for which all replicates were enriched over parent are highlighted in red. (B) Dot plot representation of variant fold change enrichment as in (A), clustered by amino acid properties (amino acid variations of residues 12 and 14 shown on the x-axis and y-axis, respectively). Note amino acid properties listed for each cluster shown refer to those for residue 12 above and to residue 14 below. *n = 3* separate pooled libraries. (C) A combinatorial library was designed to explore all amino acid combinations (excluding cysteine and methionine, library size = 324 variants) at additional residues (T12, T14) at the 53BP1/i53 interface using L67R as the parent sequence. CD34+ HSPC cells were transduced using lentivirus packaged with the combinatorial library and edited in triplicate at the *HBB* locus using HBB-UbC-GFP AAV6 (MOI = 2500). Differential variant enrichment was calculated from the variant abundance in sorted mCherry+GFP+ over mCherry+GFP- populations and graphed relative to parent L67R, shown in green. Variants with average fold change over parent (i53) larger than 1.0 are highlighted in red (15 out of 323, or 4.6%). (D) Dot plot representation of variant fold change enrichment as in (C), clustered by amino acid properties (amino acid variations of residues 12 and 14 shown on the x-axis and y-axis, respectively). Note amino acid properties listed for each cluster shown refer to those for residue 12 above and to residue 14 below. *n = 3* separate pooled libraries. (A-D) Source data are provided as a Source Data file.

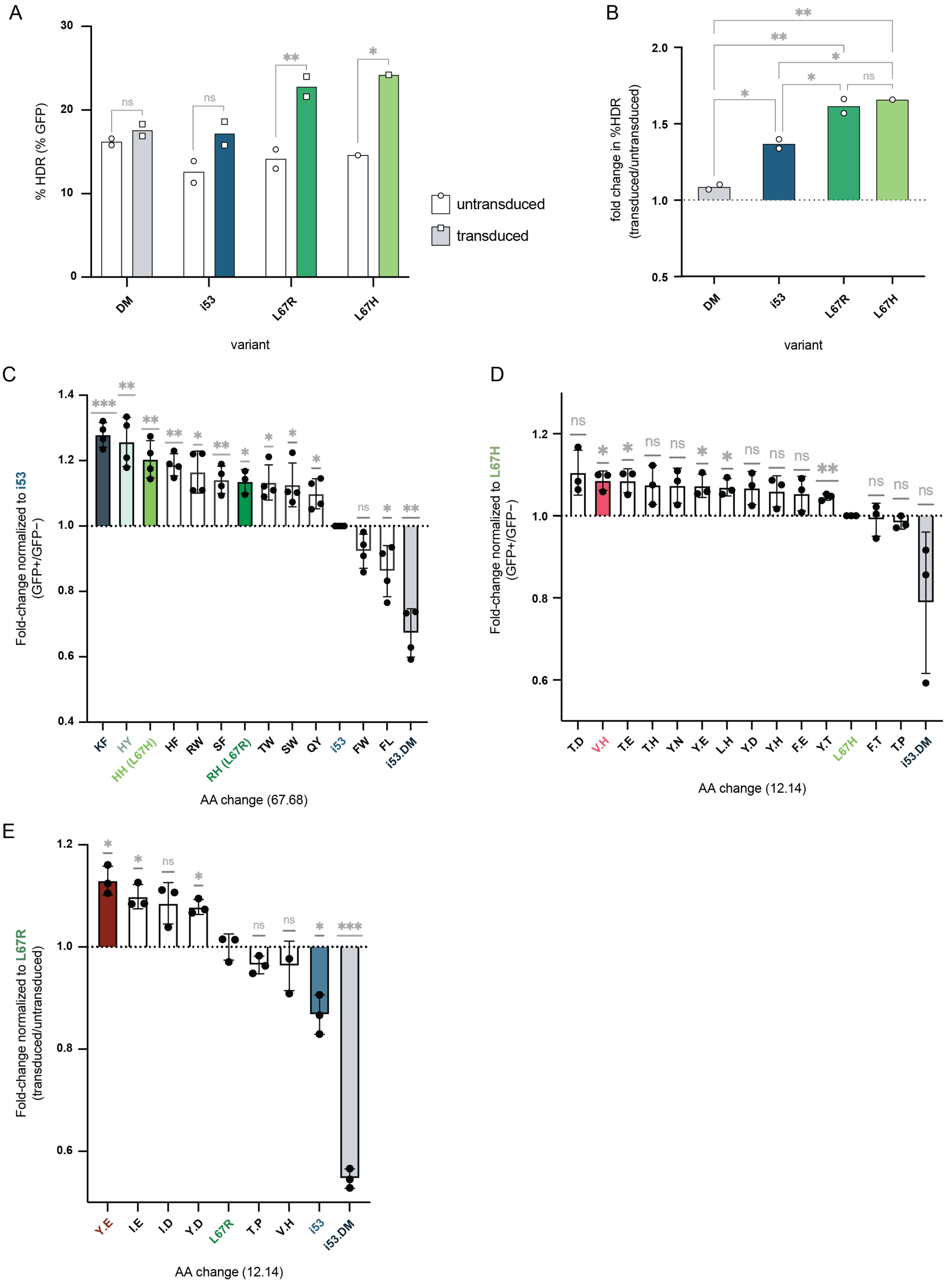

A
% HDR (% GFP)
0
10
20
30
ns
ns
**
*
DM
i53
L67R
L67H
variant
untransduced
transduced
B
fold change in %HDR
(transduced/untransduced)
0.5
1.0
1.5
2.0
**
**
*
*
*
ns
DM
i53
L67R
L67H
variant
C
Fold-change normalized to i53
(GFP+/GFP−)
0.4
0.6
0.8
1.0
1.2
1.4
***
**
**
**
*
**
*
*
*
*
ns
*
**
KF
HY
HH (L67H)
HF
RW
SF
RH (L67R)
TW
SW
QY
i53
FW
FL
i53.DM
AA change (67.68)
D
Fold-change normalized to L67H
(GFP+/GFP−)
0.6
0.8
1.0
1.2
ns
*
*
ns
ns
*
*
ns
ns
ns
**
ns
ns
ns
T.D
V.H
T.E
T.H
Y.N
Y.E
L.H
Y.D
Y.H
F.E
Y.T
L67H
F.T
T.P
i53.DM
AA change (12.14)
E
Fold-change normalized to L67R
(transduced/untransduced)
0.6
0.8
1.0
1.2
*
*
ns
*
ns
ns
*
***
Y.E
I.E
I.D
Y.D
L67R
T.P
V.H
i53
i53.DM
AA change (12.14)

***Supplementary Figure 1.5*: Validation of the top hits from the NNK (single residue) and combinatorial libraries at residues via lentiviral expression.** (A) CD34+ HSPCs were transduced to express either L67R, L67H, i53 parent or negative control i53 dead mutant (DM) and edited in duplicate at the *HBB* locus using HBB-UbC-GFP AAV6 (MOI = 1250). Flow cytometry was used to compare frequency of GFP+ positive cells in mCherry+ fraction expressing i53 variant to mCherry- fraction (no variant expression). $n = 2$ replicates across same HSPC donor and mean depicted. Analysis by one-way ANOVA with post-hoc multiple comparisons analysis. n.s. = not significant; *$p < 0.05$; **$p < 0.01$. (B) Fold change in %GFP positive cells as in (A) showing impact of variant expression on HDR-based outcomes. $n = 2$, mean depicted. Analysis by one-way ANOVA with post-hoc multiple comparisons analysis. n.s. = not significant; *$p < 0.05$; **$p < 0.01$. (C-E) Top variants identified from the various combinatorial screens were cloned individually into the lentiviral vector shown above. The resulting plasmids were either pooled together along with controls to generate small validation libraries (C, D) or used individually (E, alongside controls) to generate lentivirus. CD34+ HSPC cells were transduced with the resulting lentivirus batches and edited at the *HBB* locus using HBB-UbC-GFP AAV6 (MOI = 2500). Differential variant enrichment data shown in A and B was calculated using the fold change in the pooled library variant abundance in sorted mCherry+GFP+ and mCherry+GFP- populations (normalized to parents i53 and L67H, respectively). In order to calculate the normalized fold changes in %HDR in transduced over untransduced cells depicted in E, the rates of GFP integration in mCherry+ live cells (transduced, expressing the variant of interest) and mCherry- live cells (untransduced, control) were calculated for each replicate and normalized to the L67R average. $n = 3$ separate pooled libraries and mean ± SD depicted. Analysis by one-way ANOVA with post-hoc multiple comparisons analysis. n.s. = not significant; *$p < 0.05$; **$p < 0.01$; ***$p < 0.001$. (A-E) Source data are provided as a Source Data file.

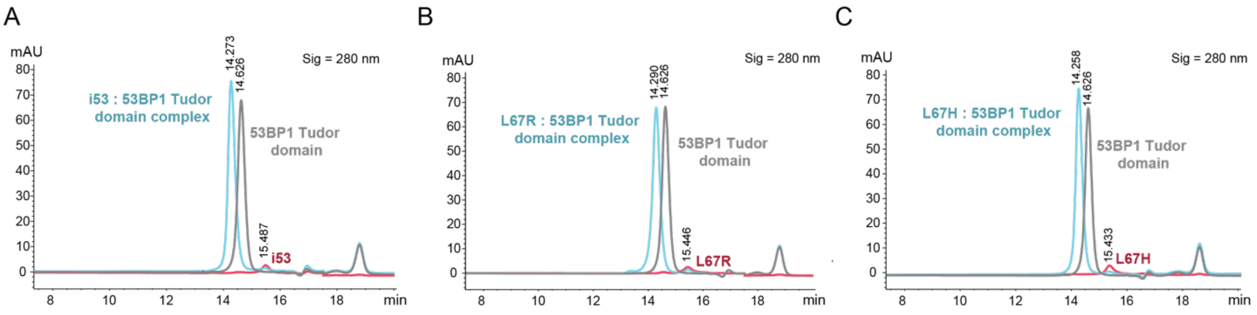

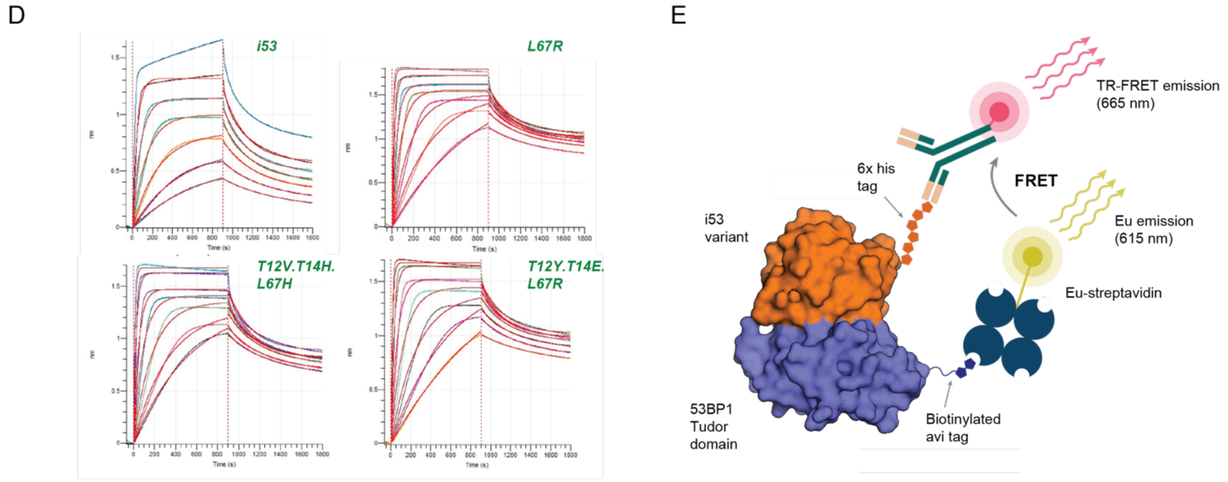

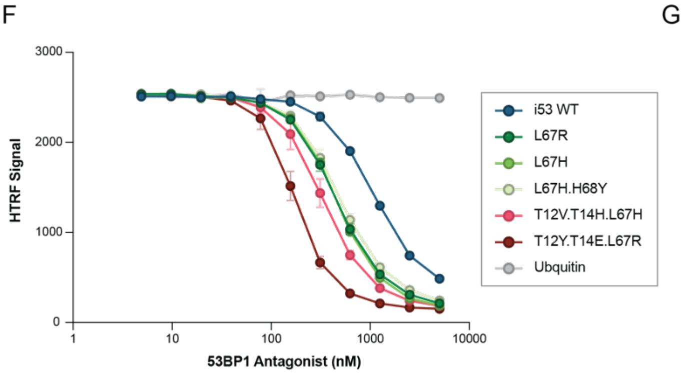

G

Protein name	*Fold increased affinity (BLI)*	*TR-FRET EC_{50} (nM ± SEM)*
i53 (WT)	1.0	1078.64 ± 22.94
L67R	2.3	458.32 ± 6.73
L67H	2.2	464.65 ± 3.79
L67H.H68Y	2.3	501.34 ± 9.32
T12Y.T14E.L67R	1.2	178.78 ± 3.85
T12V.T14H.L67H	2.0	337.73 ± 9.76
Ubiquitin	NA	NA

***Supplementary Figure 1.6*: Validation of i53 variant binding with 53BP1 Tudor Domain.** (A-C) Example Size Exclusion Chromatography traces of i53 variants (8.6 kDa; in red) and 53BP1 Tudor domain (13.9 kDa in black) and mixed variants + Tudor domain (2-fold excess of i53; in blue) are shown: (A) WT i53, (B) L67R and (C) L67H. i53 variant retention time: ~15.5 min. 53BP1 Tudor domain retention time: 14.6 min. i53 variant and 53BP1 complex retention time: 14.3 min. (D) Immobilized 53BP1 Tudor domain binding to i53 variants by Biolayer Interferometry (BLI). (E) A schematic detailing the design of a TR-FRET assay to assess binding between the i53 variants and the 53BP1 Tudor domain. (F) 53BP1 Tudor domain and i53 protein-protein interaction by TR-FRET. Source data are provided as a Source Data file. (G) Table listing the fold change in affinities between the K_D i53 variant proteins relative to K_D of i53 WT (as determined by BLI, refer to Supplementary Table S1.1 for data used to determine relative K_Ds) and TR-FRET EC_{50}s (± SEM, calculated using a non-linear 4 parameter curve fit).

Supplementary Table S1.1: **Biolayer interferometry (BLI) Data Table.**

Protein	K_D(M)	Conc (nM)	K_D (M)	ka (1/Ms)	kdis (1/s)
**i53**[a]	5.60E-08	400			
		200	5.01E-08	8.74E+04	4.38E-03
		100	4.81E-08	8.46E+04	4.07E-03
		50	4.91E-08	7.20E+04	3.53E-03
		25	7.67E-08	3.84E+04	2.94E-03
		12.5			2.27E-03
		6.25			1.75E-03
**L67R**[a]	2.42E-08	400	2.21E-08	1.98E+05	4.38E-03
		200	2.47E-08	1.81E+05	4.47E-03
		100	2.49E-08	1.72E+05	4.27E-03
		50	2.52E-08	1.56E+05	3.92E-03
		25	2.44E-08	9.87E+04	3.39E-03
		12.5			2.89E-03
		6.25			2.10E-03
**T12Y.T14E.L67R**[a]	4.60E-08	400	3.10E-08	1.47E+05	4.56E-03
		200	3.22E-08	1.30E+05	4.20E-03
		100	3.46E-08	1.15E+05	3.98E-03
		50	3.98E-08	8.85E+04	3.52E-03
		25	9.24E-08	3.29E+04	3.04E-03
		12.5			2.46E-03
		6.25			1.75E-03
**T12V.T14H.L67H**[a]	2.74E-08	400	2.42E-08	1.87E+05	4.53E-03
		200	2.74E-08	1.67E+05	4.57E-03
		100	2.61E-08	1.60E+05	4.17E-03
		50	2.69E-08	1.43E+05	3.84E-03
		25	3.25E-08	9.81E+04	3.19E-03
		12.5			2.89E-03
		6.25			2.36E-03
**i53**[b]	9.56E-08	400			
		200	7.34E-08	7.59E+04	5.57E-03
		100	8.14E-08	6.07E+04	4.94E-03
		50	1.32E-07	3.04E+04	4.04E-03
		25			3.15E-03
		12.5			1.90E-03
		6.25			1.39E-03
**L67H**[b]	4.39E-08	400	4.06E-08	1.49E+05	6.03E-03
		200	4.16E-08	1.37E+05	5.68E-03
		100	4.13E-08	1.28E+05	5.26E-03
		50	5.21E-08	8.83E+04	4.60E-03
		25			3.99E-03
		12.5			2.92E-03
		6.25			
**L67H.H68Y**[b]	4.18E-08	400	3.63E-08	1.59E+05	5.78E-03
		200	3.62E-08	1.49E+05	5.39E-03
		100	3.56E-08	1.40E+05	4.97E-03
		50	3.77E-08	1.20E+05	4.51E-03
		25	6.30E-08	6.17E+04	3.89E-03
		12.5			2.98E-03
		6.25			1.86E-03

Data obtained over two runs. [a]: Run #1. [b]: Run #2.

A

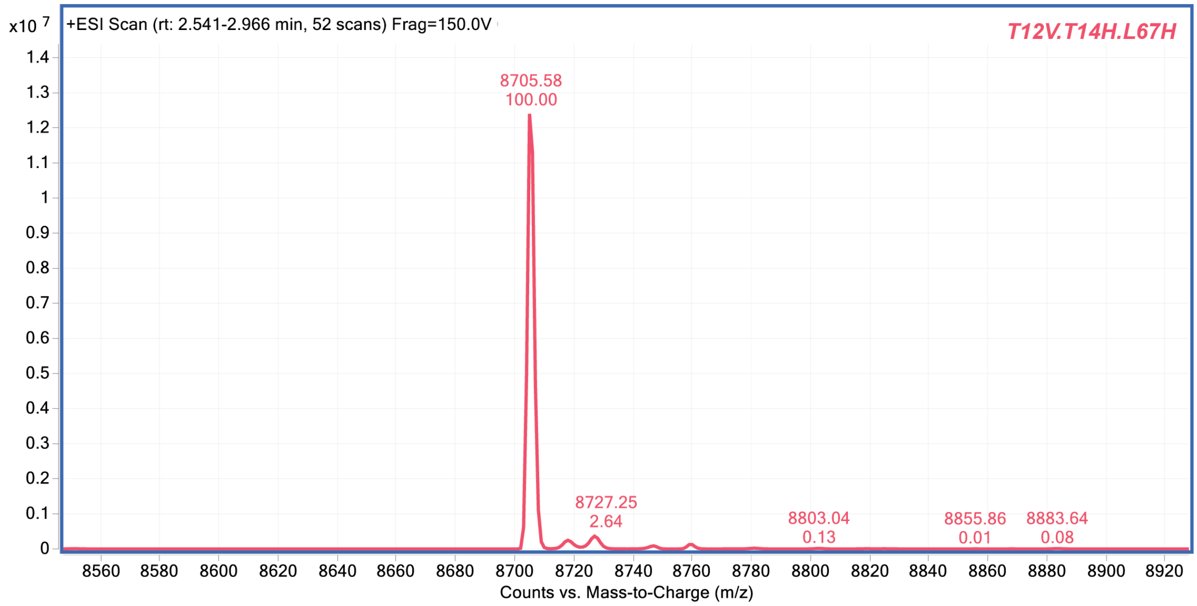

B

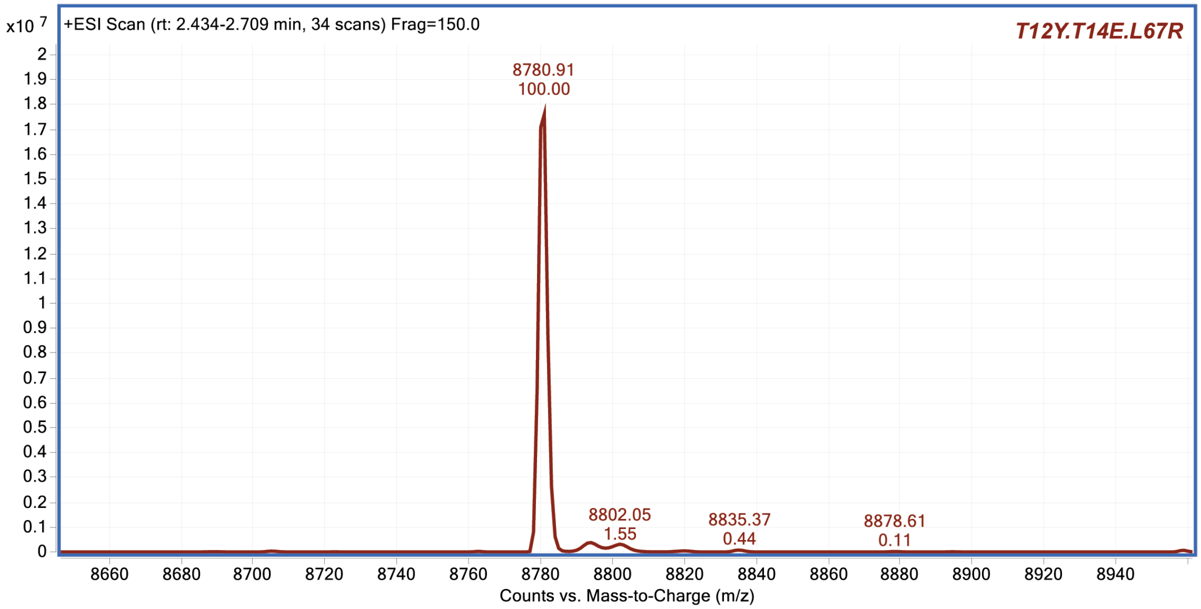

***Supplementary Figure 2.1*: Mass spectrometry traces of i53 variants T12V.T14H.L67H and T12Y.T14E.L67R.** (A) T12V.T14H.L67H (expected MW = 8705.04) and (B) T12Y.T14E.L67R (expected MW = 8780.00). Y-axis quantifies spectral counts for peptide species; X-axis shows mass-to-charge ratio (m/z). Mass spectrometry confirms mutated residues at amino acid positions 12, 14 and 67.

***Supplementary Table S2.1*: Data collection and refinement statistics (molecular replacement)**

	i53WT: 53BP1	i53L67R: 53BP1	i53L67H: 53BP1	i53T12V.T14H.L67H: 53BP1	i53T12Y.T14E.L67R: 53BP1
**Data collection**					
Space group	P21 21 21	P21 21 21	P21 21 21	P21 21 21	P21 21 21
Cell dimensions					
a, *b*, *c* (Å)	40.37 46.86 90.32	40.46 46.96 90.04	40.38 46.87 89.73	39.98 46.92 90.57	39.510 46.85 91.12
α, β, γ (°)	90, 90, 90	90, 90, 90	90, 90, 90	90, 90, 90	90, 90, 90
Resolution (Å)	1.21-25.33(1.21-1.23)	1.16-25.34 (1.16-1.18)	1.15-25.21 (1.15-1.17)	1.50-22.71 (1.50-1.53)	1.75-29.85 (1.75-1.81)
R_{sym} or R_{merge}	0.05269 (0.3571)	0.08622 (0.4187)	0.08667 (0.9862)	0.05869 (0.7245)	0.06022 (1.635)
$I / \sigma I$	18.68 (5.5)	12.0 (3.0)	10.1 (1.6)	15.9 (2.5)	12.41 (0.59)
Completeness (%)	96.93 (93.5)	99.8 (99.1)	98.0 (86)	99.8 (99.7)	98.89 (94.24)
Redundancy	7.5 (7.5)	7.0 (4.3)	7.0 (4.8)	7.3 (7.3)	6.6 (3.8)
**Refinement**					
Resolution (Å)	25.3-1.21	25.34-1.16	25-1.15	22.71-1.5	29.85 -1.75
No. reflections	51451	60078	59957	27947	17557
R_{work} / R_{free}	0.1804/0.1892	0.1977/0.2194	0.2055/0.2198	0.2096/0.2195	0.2271/0.2673
No. atoms					
Protein	1822	1810	1830	1695	1576
Ligand/ion	0	0	0	0	0
Water	241	227	187	111	38
B-factors					
Protein	15.16	16.31	13.49	32.43	45.14
Ligand/ion	N/A	N/A	N/A	N/A	N/A
Water	25.06	26.69	27.07	38.78	39.80
R.m.s. deviations					
Bond lengths (Å)	0.014	0.014	0.014	0.012	0.009
Bond angles (°)	1.95	1.93	1.87	1.82	1.53

1 crystal per dataset. *Values in parentheses are for highest-resolution shell

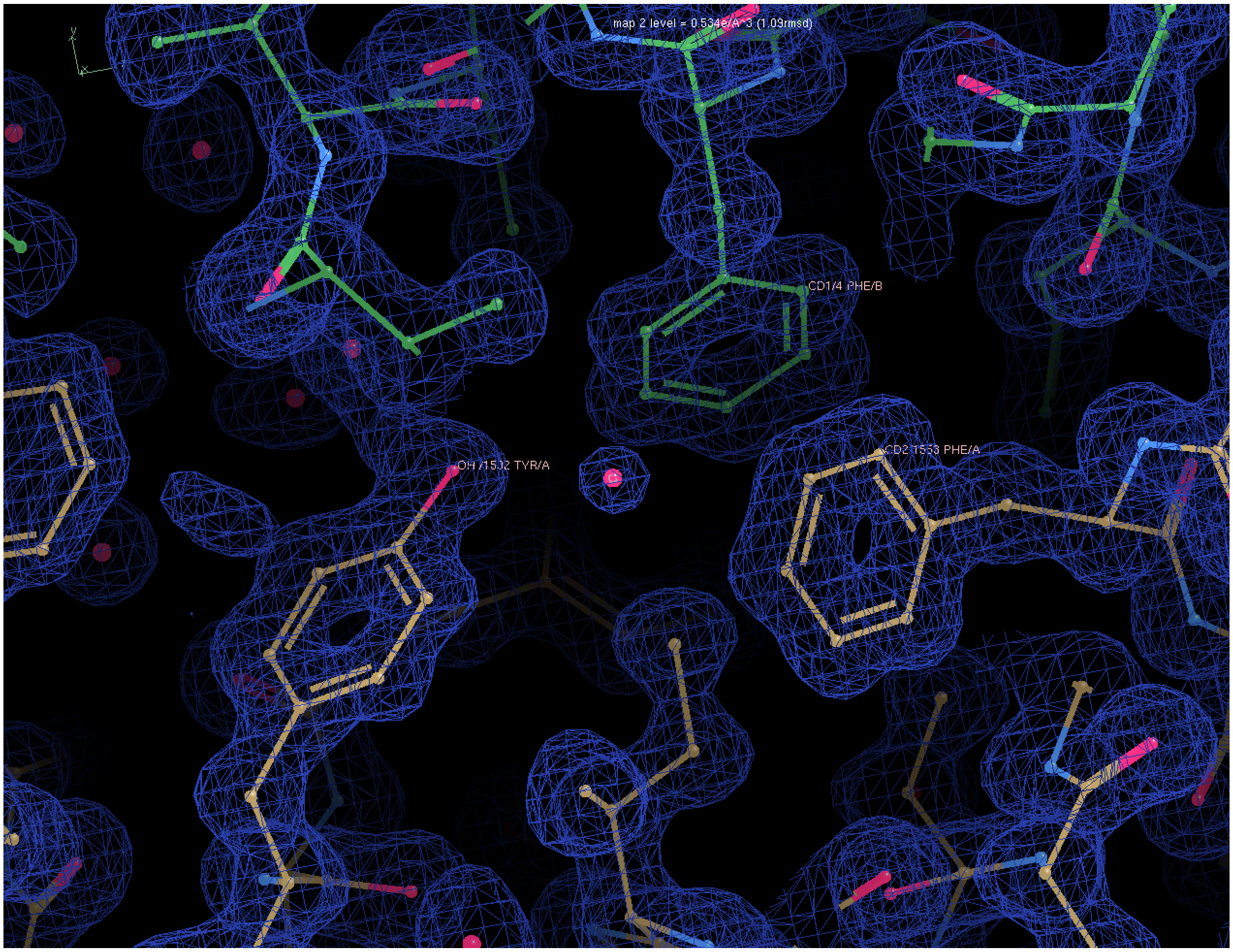

Supplementary Figure 2.2**: Representative electron density 2FO-FC map for the i53:53BP1 Tudor domain complex structure.** Contour level of 1.09 rmsd.

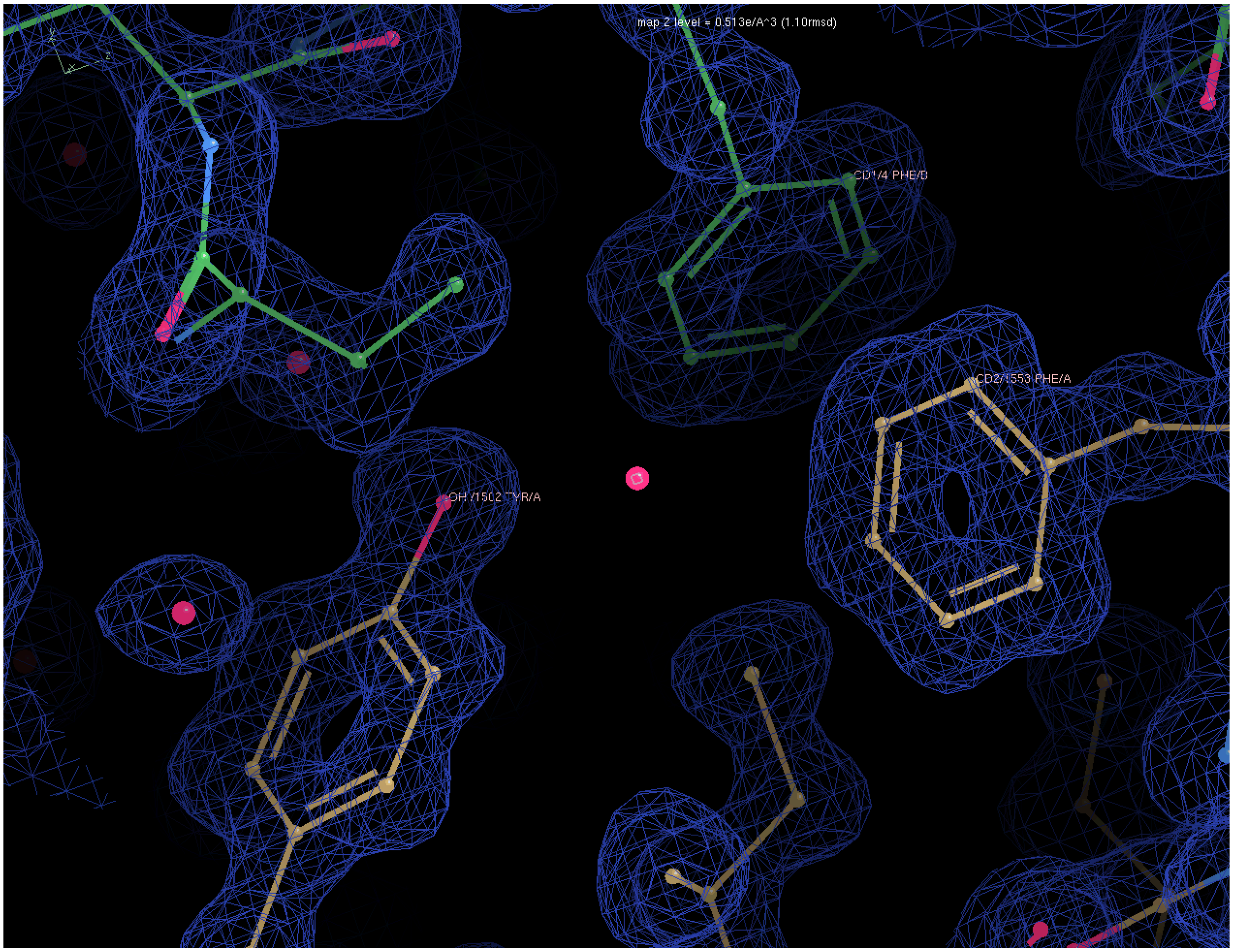

Supplementary Figure 2.3**: Representative electron density 2FO-FC map for the L67R:53BP1 Tudor domain complex structure.** Contour level of 1.10 rmsd.

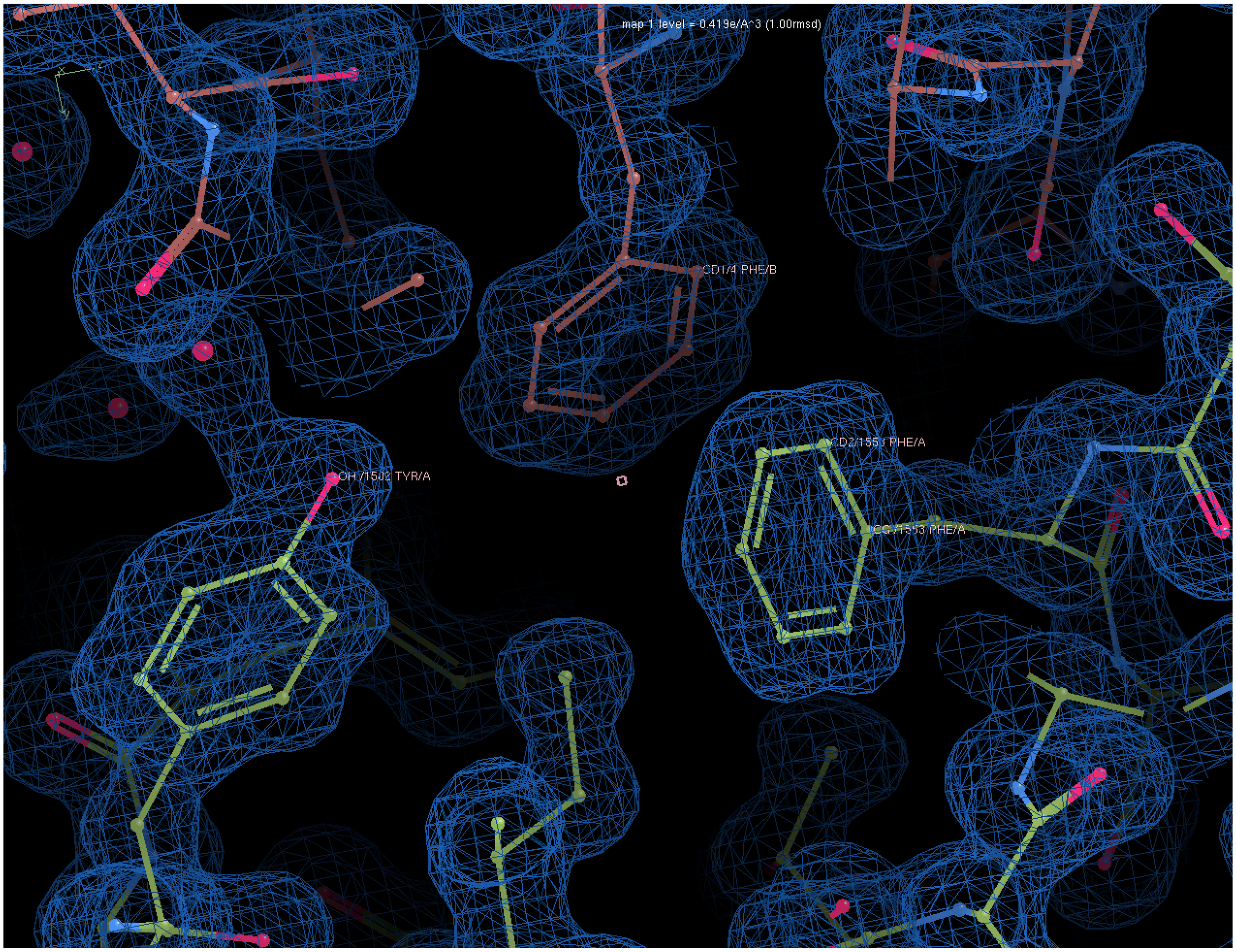

***Supplementary Figure 2.4*: Representative electron density 2FO-FC map for the L67H:53BP1 Tudor domain complex structure.** Contour level of 1.00 rmsd.

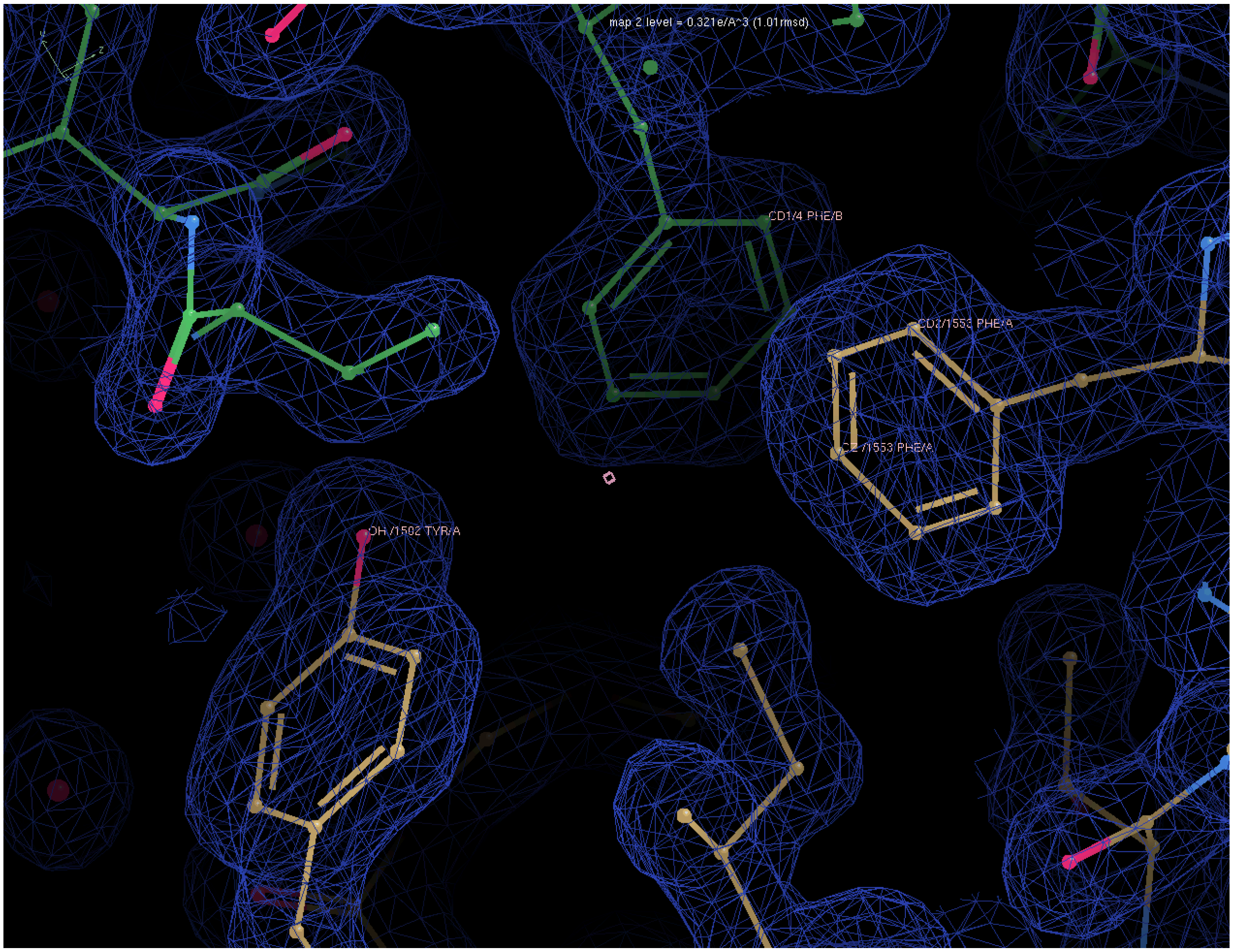

***Supplementary Figure 2.5*: Representative electron density 2FO-FC map for the T12V.T14H.L67H:53BP1 Tudor domain complex structure.** Contour level of 1.01 rmsd.

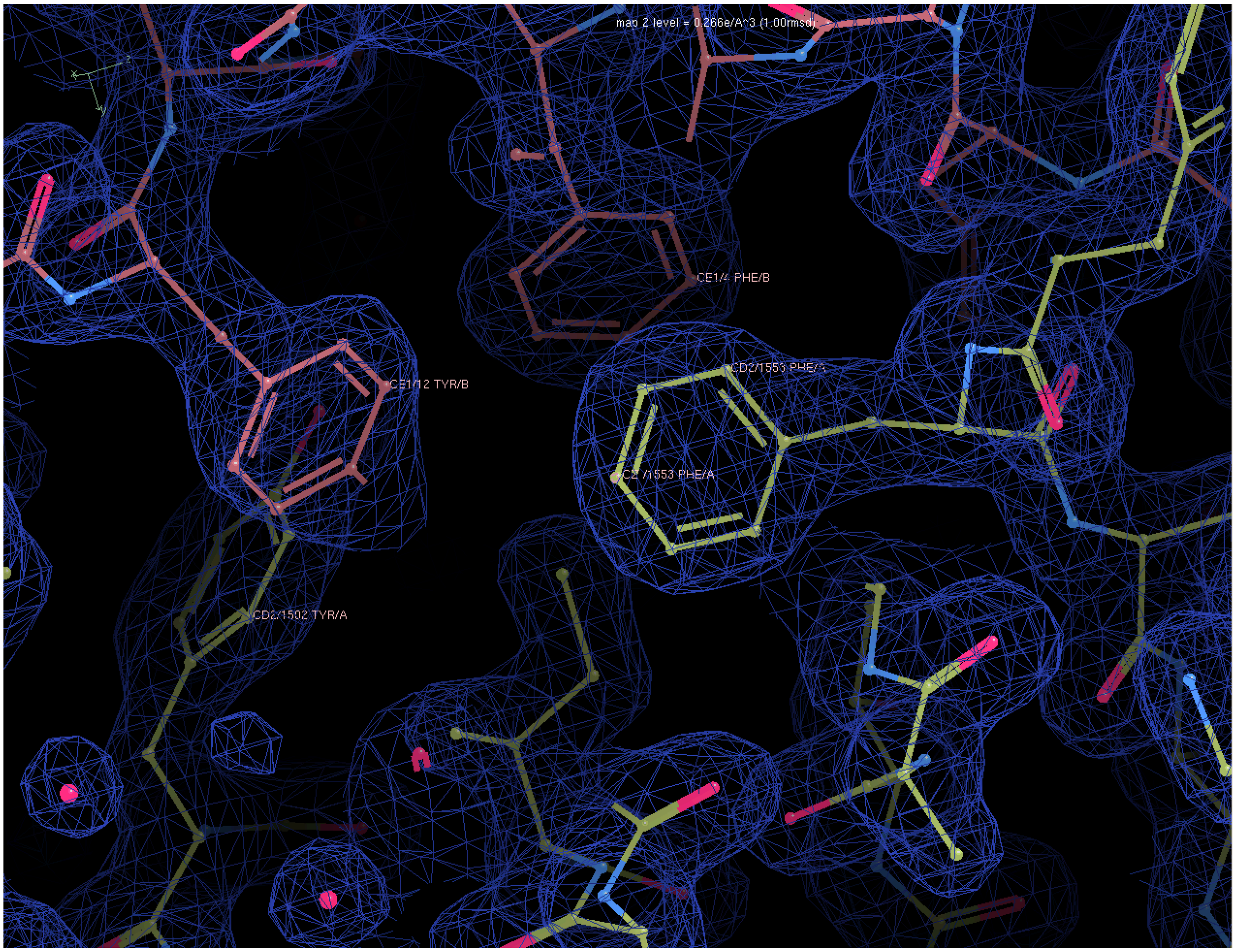

***Supplementary Figure 2.6*: Representative electron density 2FO-FC map for the T12Y.T14E.L67R:53BP1 Tudor domain complex structure.** Contour level of 1.00 rmsd.

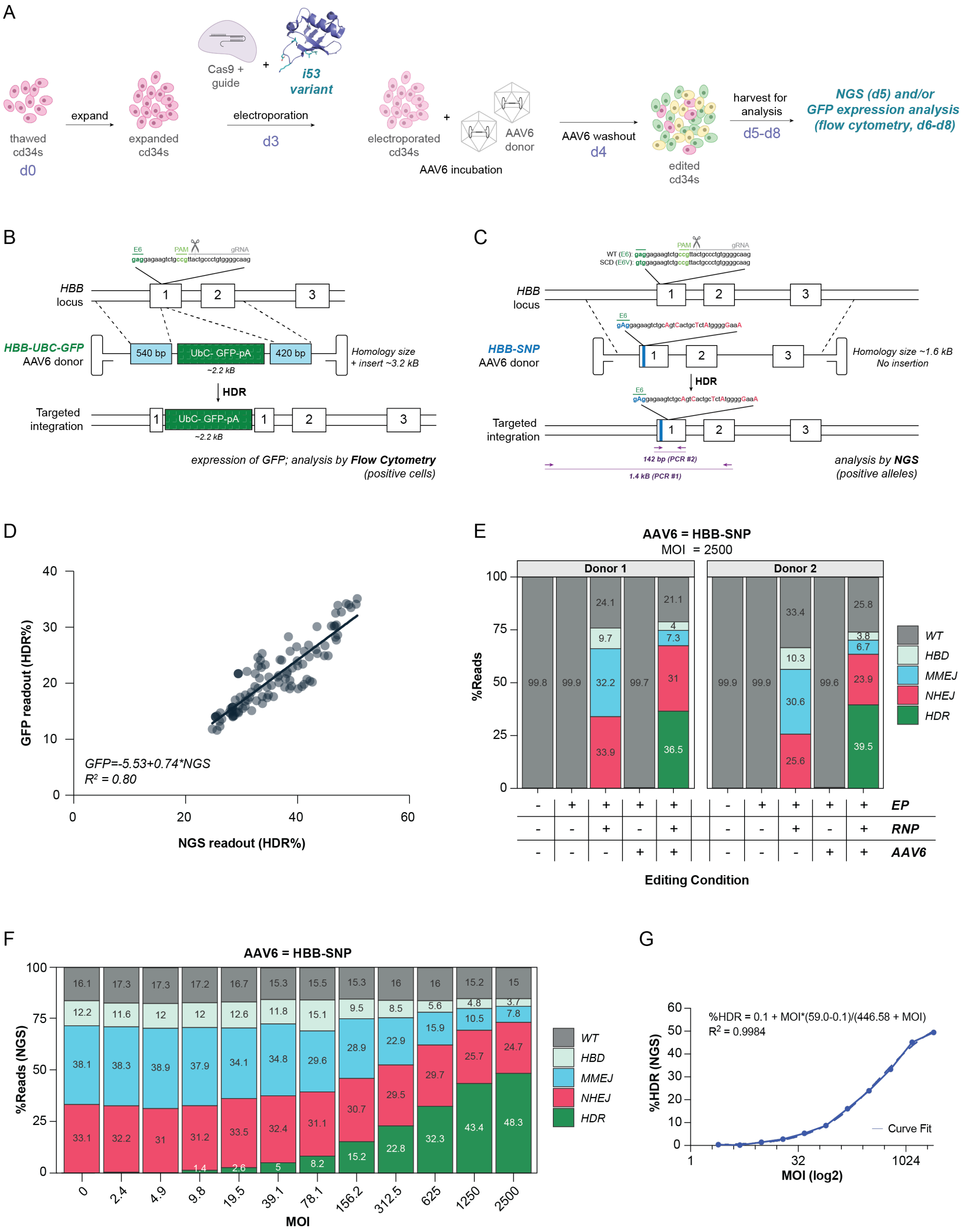

A
Cas9 + guide
i53 variant
thawed cd34s
d0
expand
expanded cd34s
electroporation
d3
electroporated cd34s
AAV6 donor
AAV6 incubation
AAV6 washout
d4
edited cd34s
harvest for analysis
d5-d8
NGS (d5) and/or GFP expression analysis (flow cytometry, d6-d8)
B
HBB locus
HBB-UBC-GFP AAV6 donor
540 bp
UbC- GFP-pA
420 bp
~2.2 kB
Homology size + insert ~3.2 kB
HDR
Targeted integration
expression of GFP; analysis by Flow Cytometry (positive cells)
C
HBB locus
HBB-SNP AAV6 donor
Homology size ~1.6 kB No insertion
HDR
Targeted integration
142 bp (PCR #2)
1.4 kB (PCR #1)
analysis by NGS (positive alleles)
D
GFP readout (HDR%)
NGS readout (HDR%)
GFP=-5.53+0.74*NGS
R² = 0.80
E
AAV6 = HBB-SNP
MOI = 2500
Donor 1
Donor 2
%Reads
WT
HBD
MMEJ
NHEJ
HDR
EP
RNP
AAV6
Editing Condition
F
AAV6 = HBB-SNP
%Reads (NGS)
MOI
WT
HBD
MMEJ
NHEJ
HDR
G
%HDR = 0.1 + MOI*(59.0-0.1)/(446.58 + MOI)
R² = 0.9984
%HDR (NGS)
MOI (log2)
Curve Fit

Supplementary Figure 3.1: **Protocol for analysis of editing outcomes used in this study.** (A) Schematic detailing the procedure for CD34+ HSPC editing using Cas9 RNP and an AAV6, including incorporation of the purified i53 variant protein additives. Editing occurs 3 days post CD34+ HSPC cell thaw; purified i53 variants are incorporated at various concentrations into the nucleofection solution containing Cas9 and guide RNA. After nucleofection, the cells are incubated in media containing various concentrations of AAV6 for 24 h and harvested for analysis 24 – 96 hours post AAV6 washout. (B-C) Schematic representation of the two editing approaches used for the *HBB* locus. An AAV template is used for HDR repair, containing either (B) a knock-in cassette expressing GFP (readout is % GFP positive cells by flow cytometry, reports HDR only, used in different alleles) or (C) a homology template bearing single nucleotide changes (readout is % alleles by NGS, reports all editing outcomes, *HBB* locus only). (D) Correlation between HDR efficiencies obtained using the GFP insertion method and the HBB-SNP method, for matching samples (editing in parallel in identical conditions; incubated in different AAV templates). The GFP knock-in procedure requires a large insertion so efficiency is generally lower than that of the HBB-SNP procedure. *n = 108* datapoints across 4 different editing experiments and 6 different HSPC donors. (E) Editing outcomes analyzed by NGS when different control conditions are used. *n = 2* donors, displayed separately; average of 2 technical replicates. (F-G) Editing outcomes analysis for a titration of AAV template concentrations, illustrating the dynamic range of the assay. *n = 1* HSPC donor; average of 2 technical replicates. Contributions below 1% are not shown. (D-G) Source data are provided as a Source Data file.

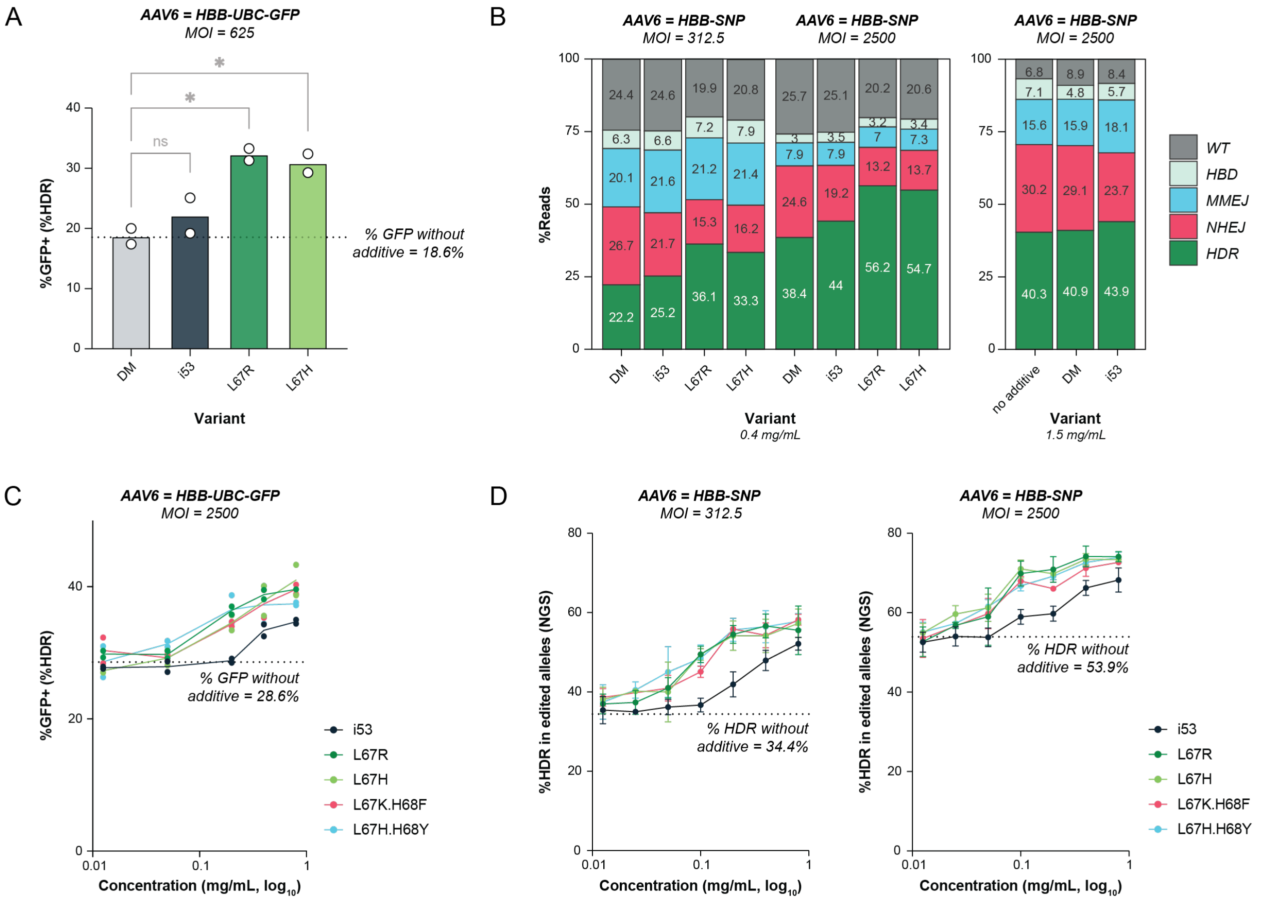

***Supplementary Figure 3.2*: Initial assessment of purified i53 variants when incorporated as protein-based additives to HSPC editing protocols.** (A) %GFP positive cells in HSPCs edited using HBB-UbC-GFP AAV6 and purified L67R, L67H, i53 parent, or negative control i53 dead mutant (DM). Variants were added to the nucleofection solutions at concentration of 0.4 mg/mL (*N* = 2 replicates for the same CD34+ HSPC donor, 7.5e5 cells/cuvette, split across 3 experimental conditions, mean is depicted). Analysis by one-way ANOVA with post-hoc multiple comparisons analysis. n.s. = not significant; $*p < 0.05$. (B) Editing outcomes at the *HBB* cut site as detected by NGS analysis in cells edited using HBB-SNP AAV6 and purified L67R, L67H, i53 parent, or negative control i53 dead mutant (DM). Variants were added to the nucleofection solutions at concentration of 0.4 or 1.5 mg/mL (*n*-= 2 replicates for the same CD34+ HSPC donor, 7.5e5 cells/cuvette, split across 3 experimental conditions; cells collected for NGS 2 d post nucleofection, mean is shown). (C) %GFP positive cells in HSPCs edited with HBB-UbC-GFP AAV6 and representative purified hit i53 variants from libraries targeting residues 67 and 68. Proteins, along with an i53 control, were added to the nucleofection solutions at concentrations of 0.0125, 0.05, 0.2, 0.4, and 0.8 mg/mL (*n* = 2 replicates for the same CD34+ HSPC donor; cells collected for NGS 2 d post nucleofection). (D) %HDR (NGS) of edited alleles in HSPCs edited using HBB-SNP AAV6 at two different MOIs; protein variants were added to the nucleofection solutions at concentrations of 0.0125, 0.025, 0.05, 0.1, 0.2, 0.4, and 0.8 mg/mL (*n* = 4 across 3 CD34+ HSPC donors, $5\text{-}7 \times 10^5$ cells/cuvette, split across 3 experimental conditions; cells collected for NGS 2 d post nucleofection, mean ± SD depicted). (A-D) Source data are provided as a Source Data file.

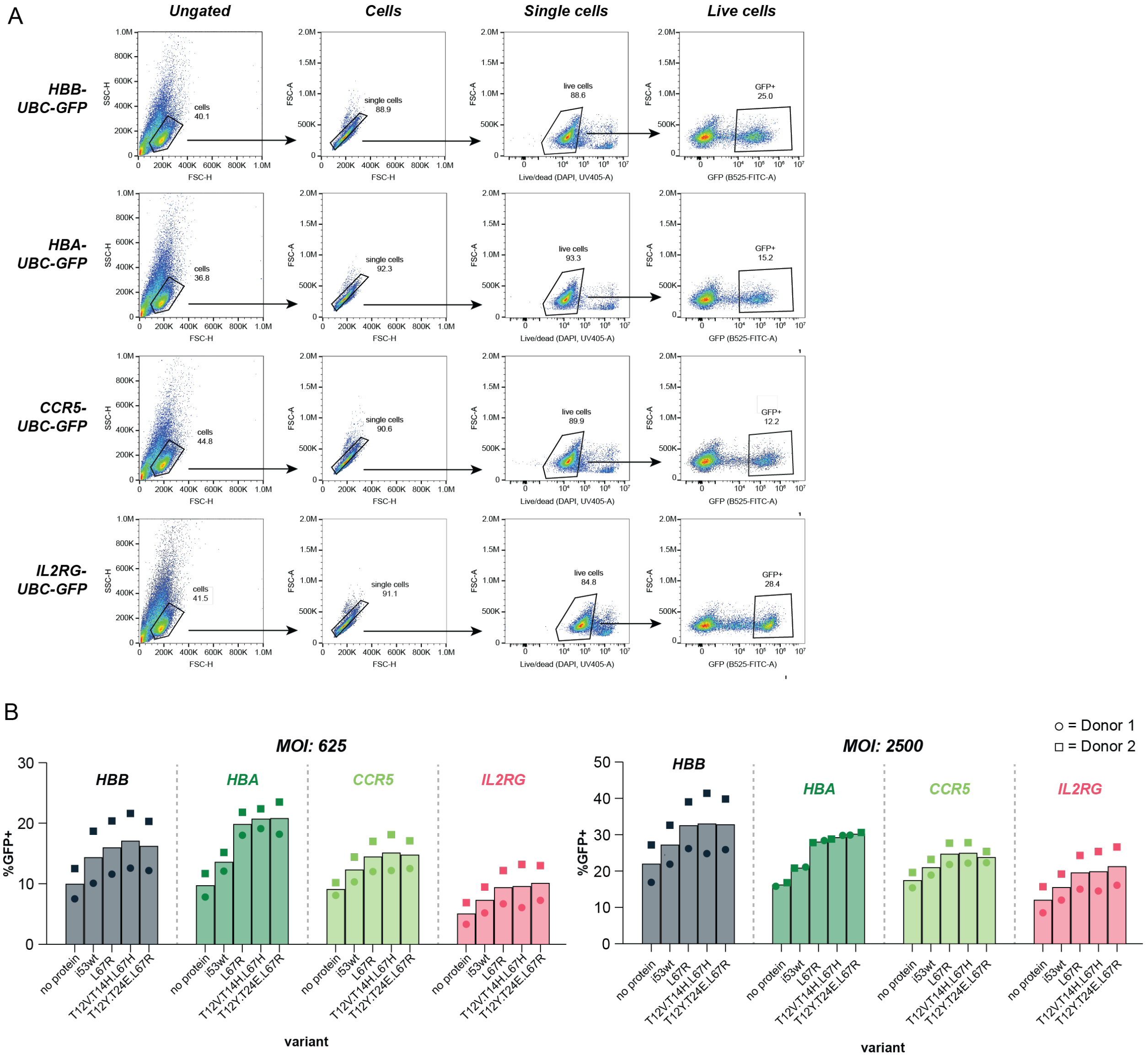

Supplementary Figure 3.3 **(relative to Figure 3A): Editing at different clinically relevant loci using GFP-encoding AAV6 and purified i53 variant proteins.** (A) Representative flow cytometry plots showing the gating strategy to isolate Single-Live GFP+ expressing cells edited with UbC-GFP AAV6 targeting multiple clinically relevant loci (*HBB*, *HBA*, *CCR5*, and *IL2RG*). (B) GFP knock-in to CD34+ HSPC cells using GFP-encoding AAV6 targeted to *HBB*, *HBA, CCR5*, and *IL2RG* loci and representative purified “hit” variants of i53 identified in the above screens (L67R, T12Y.T14E.L67R, and T12V.T14H.L67H). Protein variants, as well as a parental control WT i53, were incorporated into nucleofection solutions at concentrations of 0.4 mg/mL; post nucleofection, the cells were incubated with the AAV6 at MOIs of 625 and 2500. Cells were analyzed via flow cytometry 4 d post nucleofection; %GFP of live cells is shown. *n* = 2 separate HSPC donors (both male, $7.5x10^5$ cells/cuvette, distributed into two MOI conditions). Mean values are depicted. Source data are provided as a Source Data file.

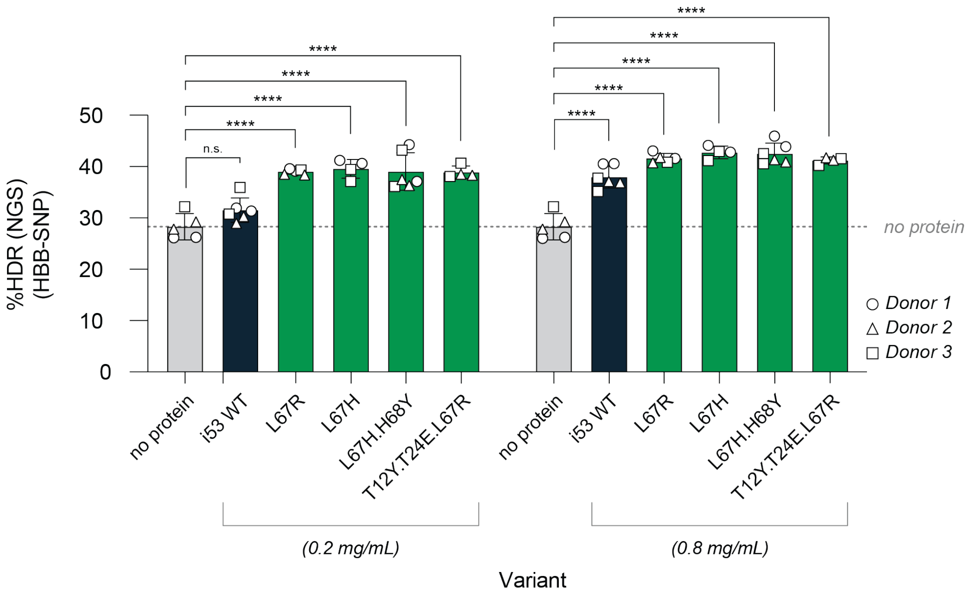

Supplementary Figure 3.4 (related to Figure 3B): **Comparison of editing efficiencies to the baseline condition (no additive).** %HDR (NGS) in HSPCs at *HBB* when edited using sickle-cell correcting HBB-SNP AAV6 (MOI = 312.5) and purified variants of i53 at two different protein concentrations. Comparisons are shown relative to no protein controls. Three different HSPC donors were used across three separate experiments (5-7x10^5 cells/cuvette). For variants i53 and L67H.H68Y, *n* = 6 and for variant L67R and no protein conditions, *n* = 5 (using three HPSC donors). For variants L67H and T12Y.T14E.L67R, *n* = 4 (using two HSPC donors). Mean ± SD depicted. Two-way ANOVA with Dunnett correction for multiple comparisons. n.s. = non-significant; ****$p < 0.0001$. Cells collected for NGS 2 d post-nucleofection. Source data are provided as a Source Data file.

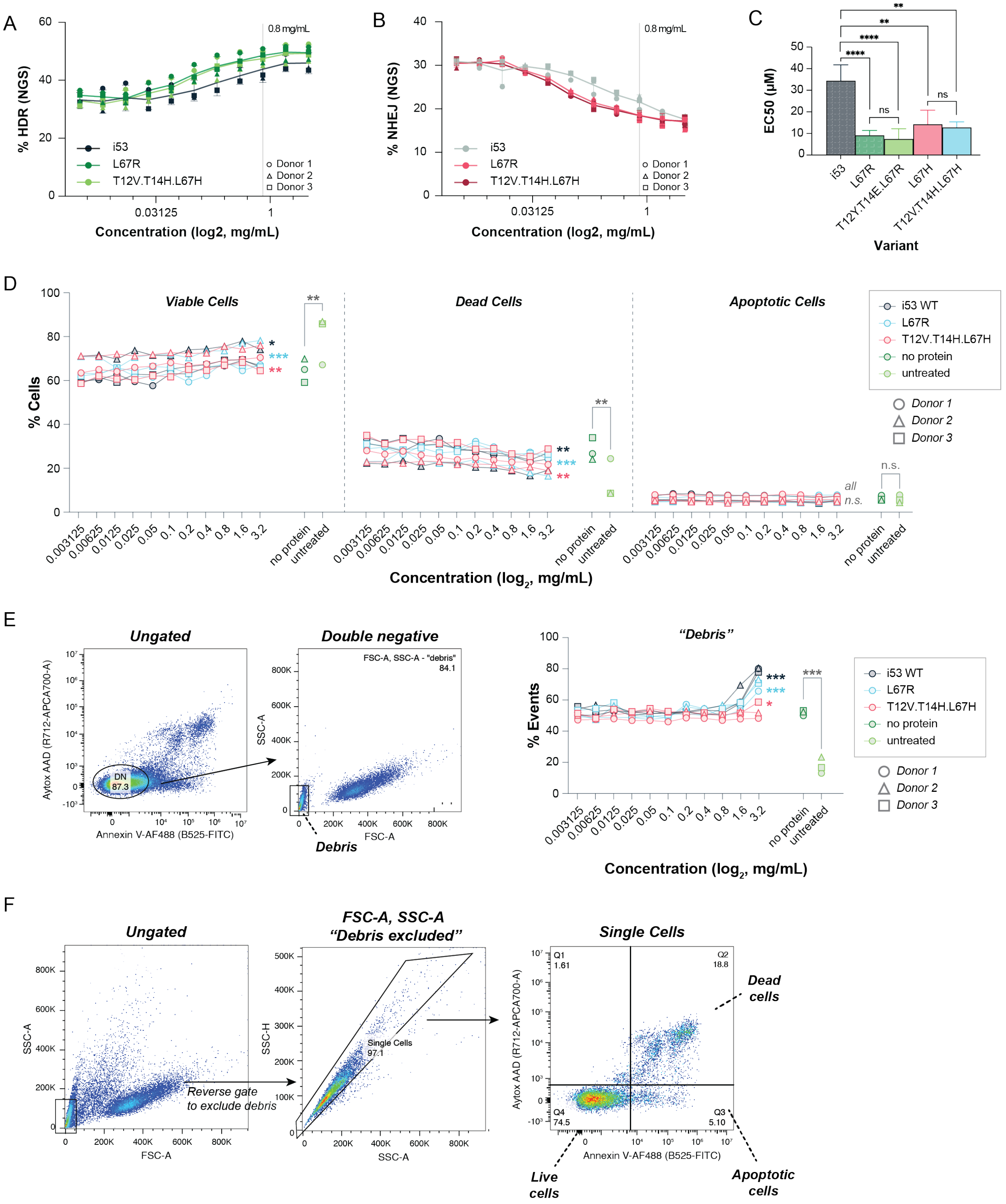

A
% HDR (NGS)
i53
L67R
T12V.T14H.L67H
0.8 mg/mL
Donor 1
Donor 2
Donor 3
Concentration (log2, mg/mL)
B
% NHEJ (NGS)
i53
L67R
T12V.T14H.L67H
0.8 mg/mL
Donor 1
Donor 2
Donor 3
Concentration (log2, mg/mL)
C
EC50 (µM)
i53
L67R
T12Y.T14E.L67R
L67H
T12V.T14H.L67H
Variant
ns
ns
D
Viable Cells
Dead Cells
Apoptotic Cells
% Cells
i53 WT
L67R
T12V.T14H.L67H
no protein
untreated
Donor 1
Donor 2
Donor 3
all n.s.
n.s.
Concentration (log2, mg/mL)
E
Ungated
Aytox AAD (R712-APCA700-A)
Annexin V-AF488 (B525-FITC)
DN 87.3
Double negative
FSC-A, SSC-A - "debris" 84.1
SSC-A
FSC-A
Debris
"Debris"
% Events
i53 WT
L67R
T12V.T14H.L67H
no protein
untreated
Donor 1
Donor 2
Donor 3
Concentration (log2, mg/mL)
F
Ungated
SSC-A
FSC-A
Reverse gate to exclude debris
FSC-A, SSC-A "Debris excluded"
SSC-H
SSC-A
Single Cells 97.1
Single Cells
Aytox AAD (R712-APCA700-A)
Annexin V-AF488 (B525-FITC)
Q1 1.61
Q2 18.8
Q3 5.10
Q4 74.5
Dead cells
Live cells
Apoptotic cells

Supplementary Figure 3.5 **(related to Figure 3C): Editing outcomes and cellular apoptosis assay on a full dose response of i53 variants using *HBB*-targeting AAV6**. Dose response curves of i53, L67R, and T12V.T14H.L67H using HBB-SNP AAV6 (MOI = 625, *n* = 3 CD34+ HSPC donors; cells collected for NGS 2 d post nucleofection) showing as absolute HDR/NHEJ values rather than fold changes. Effects of i53 variant concentration on (A) %HDR and the corresponding effects on (B) %NHEJ of NGS reads are shown. The vertical dotted line indicates the typical working concentration (0.8 mg/mL). *n* = 3 different HSPC donors. Error bars represent mean +/- SD. (C) EC50s (shown in μM) for additional i53 variants when edited using HBB-UbC-GFP and flow cytometry readout. *n* = 3 separate HSPC donors. Four-parameter dose response curve fit, using fold change relative to no additive (0 mg/mL). (D) Fraction of viable, apoptotic, or necrotic cells, 3 days after editing for each condition. *n* = 3 separate HSPC donors. For dose series, analysis was performed by linear regression (test if slope is different from zero). Control comparison by one-way ANOVA with post-hoc pairwise comparison. *$p < 0.05$; **$p < 0.01$; ***$p < 0.001$; n.s.: not significant. Note that, although significant, the effect of increasing amounts of additive in cell viability (more viable cells, less necrotic cells) was rather modest (an extra ~1.7% viable cells per 1mg/mL increase in dose). (E-F) Representative flow cytometry plots showing the gating strategy. (E) Events classified as "debris" (negative for both Annexin V and Live/Dead stain, Sytox AAD, and that have small size in both SSC-A and FSC-A) are gated out of the original population. Fraction of events classified as “debris” for each condition is shown. Dose-response analysis was performed as in panel D. Comparison of no protein and untreated conditions by two-tailed t-test. (F) After applying a reverse gate to exclude “debris,” single cells are characterized as live (negative for both Annexin V and Sytox AAD), necrotic (positive for both Annexin V and Sytox AAD), or apoptotic (positive to Annexin V, negative for Sytox AAD). (A-E) Source data are provided as a Source Data file.

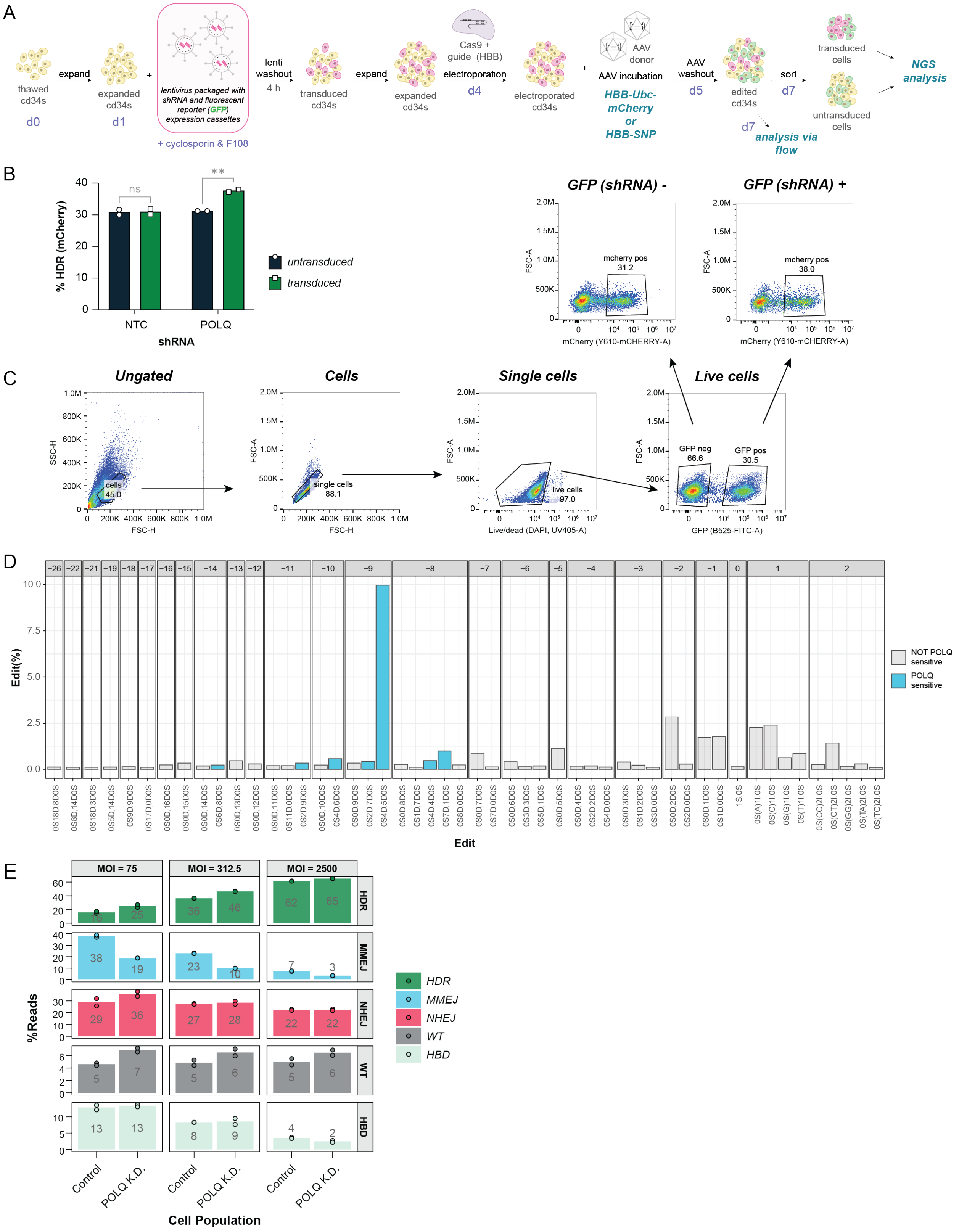
A
thawed cd34s
d0
expand
expanded cd34s
d1
lentivirus packaged with shRNA and fluorescent reporter (GFP) expression cassettes
+ cyclosporin & F108
lenti washout
4 h
transduced cd34s
expand
expanded cd34s
Cas9 + guide (HBB)
electroporation
d4
electroporated cd34s
AAV donor
AAV incubation
HBB-Ubc-mCherry
or
HBB-SNP
AAV washout
d5
edited cd34s
d7
analysis via flow
sort
d7
transduced cells
untransduced cells
NGS analysis
B
% HDR (mCherry)
ns
**
NTC
POLQ
shRNA
untransduced
transduced
GFP (shRNA) -
GFP (shRNA) +
mcherry pos
31.2
mcherry pos
38.0
FSC-A
mCherry (Y610-mCHERRY-A)
C
Ungated
Cells
Single cells
Live cells
SSC-H
FSC-H
cells
45.0
single cells
88.1
live cells
97.0
Live/dead (DAPI, UV405-A)
GFP neg
66.6
GFP pos
30.5
GFP (B525-FITC-A)
D
Edit(%)
Edit
NOT POLQ sensitive
POLQ sensitive
E
MOI = 75
MOI = 312.5
MOI = 2500
HDR
MMEJ
NHEJ
WT
HBD
%Reads
Control
POLQ K.D.
Cell Population

***Supplementary Figure 3.6*: Knockdown of *POLQ* elucidates MMEJ related outcomes in *HBB* locus editing.** (A) A schematic detailing the use of shRNA-encoding lentivirus (fluorescently tagged with GFP) to probe the impact of knocking down a gene of interest on HDR-based outcomes at *HBB* in CD34+ HSPCs using HBB-mCherry AAV6 or HBB-SNP DNA donors. Transduced cells edited with HBB-mCherry AAV6 were analyzed by flow cytometry; cells edited with HBB-SNP AAV6 were sorted (GFP+/GFP-) and analyzed via NGS. (B) shRNA expression was monitored by GFP expression and HDR frequency was measured by assessing mCherry positive cells in cell populations that expressed POLQ-targeting shRNA, a non-targeting control (NTC) shRNA, or no shRNA. n = 2 editing experiments using the same HSPC donor ($5x10^5$ cells/cuvette) and mean values are depicted. Analysis by one-way ANOVA with post-hoc pairwise comparisons and multiple testing correction. Source data are provided as a Source Data file. (C) Representative flow cytometry plots showing the gating strategy used to determine mCherry positive cells in transduced (GFP+) and un-transduced (GFP-) populations. (D) Individual INDEL editing outcomes at *HBB* and sensitivity to POLQ knockdown (HDR/WT/HBD outcomes excluded, only indels with >0.1% reads shown). Bar height represents the specific indel contribution, and fill represents if the reduction in the MMEJ knockdown was statistically significant. Using amplicon sequencing data. $n = 2$ separate editing experiments using the same HSPC donor ($1.8x10^6$ cells/cuvette, split across 3 MOI conditions). Deemed significant if p–< 0.05 using one-way ANOVA with post-hoc pairwise testing and multiple testing correction. Source data are provided as a Source Data file. (E) Editing outcome distribution when implementing MMEJ classification. *HBB* editing outcomes sensitive to POLQ knockdown are designated “MMEJ”, whereas indels that did not change in MMEJ knockdown are classified as “NHEJ”. The impact of POLQ knockdown on MMEJ edits, as well as the other categories of edits, are shown. Source data are provided as a Source Data file.

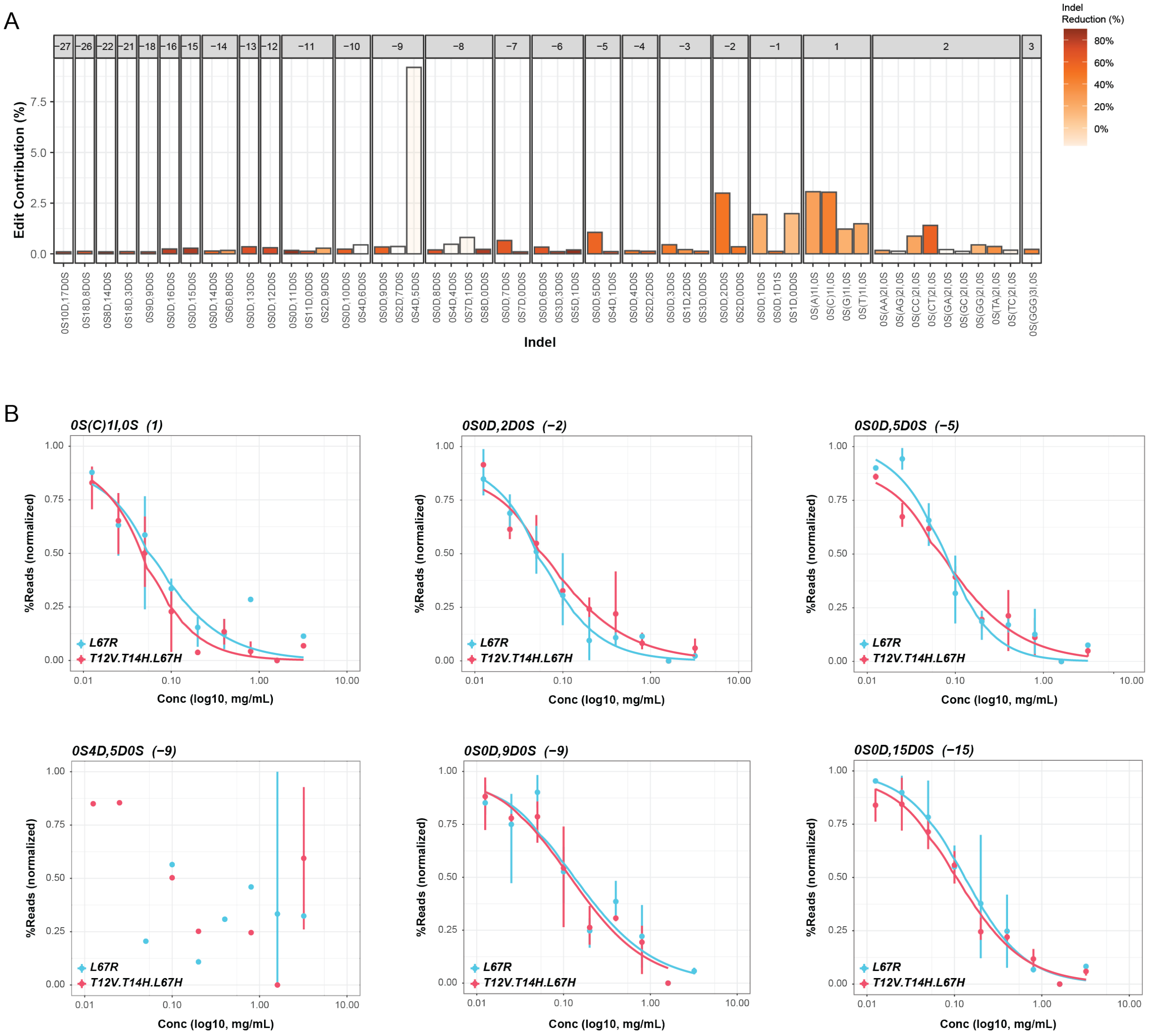

***Supplementary Figure 3.7*: Identification of 53BP1i-sensitive INDELs at the *HBB* locus cutsite. (A) Plot of the contribution of individual INDEL outcomes.** Bar shading represents normalized reduction of the prevalence of each indel when i53 variants are added for editing (HDR/WT/HBD excluded, only indels with >0.1% reads shown). White fill represent edits that did not result in a dose-dependent reduction of contribution when i53 variants were used. n = 3 different HSPC donors; each one of them with two different i53 variants at 0.8 mg/mL. An individual dose-response curve (four parameters, nonlinear) was fit for each variant and only those with significant association (p < 0.01) were categorized as i53 responsive. (B) Sample plots of the contribution of specific indels at increasing concentrations of two of the i53 variants identified in this study, along with fitted dose-response curves. An MMEJ edit (-9, bottom left) is shown as an example of a non-i53 responsive edit). n = 3 separate donors edited. (A-B) Source data are provided as a Source Data file.

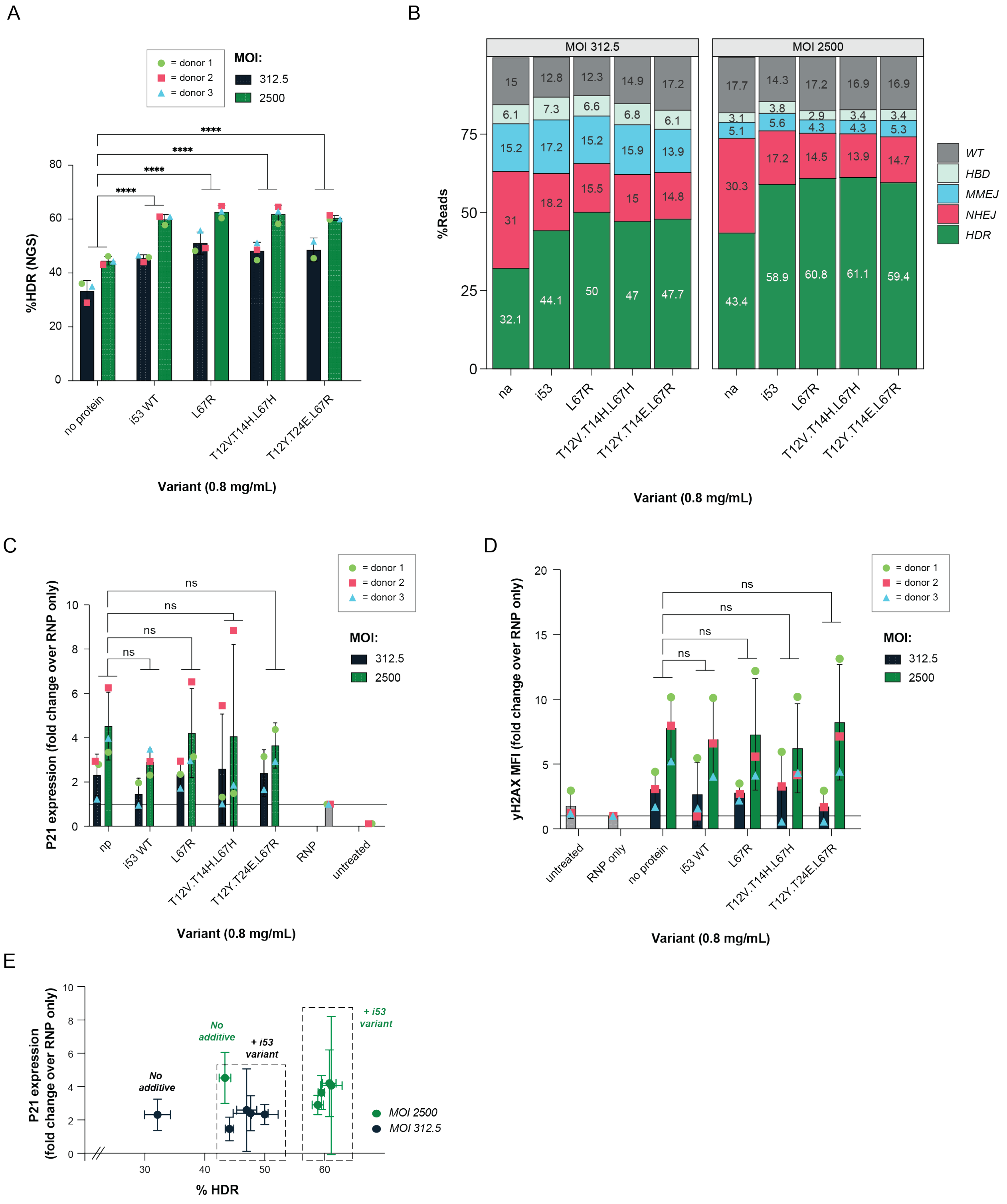

A
= donor 1
= donor 2
= donor 3
MOI:
312.5
2500
****
%HDR (NGS)
no protein
i53 WT
L67R
T12V.T14H.L67H
T12Y.T24E.L67R
Variant (0.8 mg/mL)
B
MOI 312.5
MOI 2500
%Reads
WT
HBD
MMEJ
NHEJ
HDR
na
i53
L67R
T12V.T14H.L67H
T12Y.T14E.L67R
Variant (0.8 mg/mL)
C
P21 expression (fold change over RNP only)
ns
np
i53 WT
L67R
T12V.T14H.L67H
T12Y.T24E.L67R
RNP
untreated
Variant (0.8 mg/mL)
D
yH2AX MFI (fold change over RNP only)
untreated
RNP only
no protein
i53 WT
L67R
T12V.T14H.L67H
T12Y.T24E.L67R
Variant (0.8 mg/mL)
E
P21 expression (fold change over RNP only)
No additive
+ i53 variant
MOI 2500
MOI 312.5
% HDR

Supplementary Figure 3.8 **(related to Figure 3D and 3E): Editing of HSPCs using HBB-SNP AAV6 and purified i53 variant proteins.** (A-B) HSPCs edited using HBB-SNP AAV6 and purified variants of i53 (MOI = 312.5 and 2500, *N* = 3; cells collected for NGS 2 d post nucleofection). (A) %HDR and (B) averaged %reads for the different *HBB* editing outcomes as determined by NGS analysis. *n* = 3 different HSPC donors (2x10^6 cells/cuvette, split across 2 MOI conditions) and mean +/- SD depicted. Analysis by two-way ANOVA with post-hoc pairwise comparison and multiple testing correction. Only showing the variant effect. ***p < .0001. (C-D) Induction of the DNA Damage Response (DDR) as measured by (C) expression of P21 and (D) phosphorylation of histone H2AX (yH2AX) 24 hours post nucleofection. *n = 3* different HSPC donors and mean +/- SD depicted. Analysis by two-way ANOVA with post-hoc pairwise comparison and multiple testing correction. Only showing the variant effect. n.s.: not significant. (E) %HDR relative to P21 expression with and without the addition of the i53 variants. Mean +/- SEM depicted. (A-E) Source data are provided as a Source Data file.

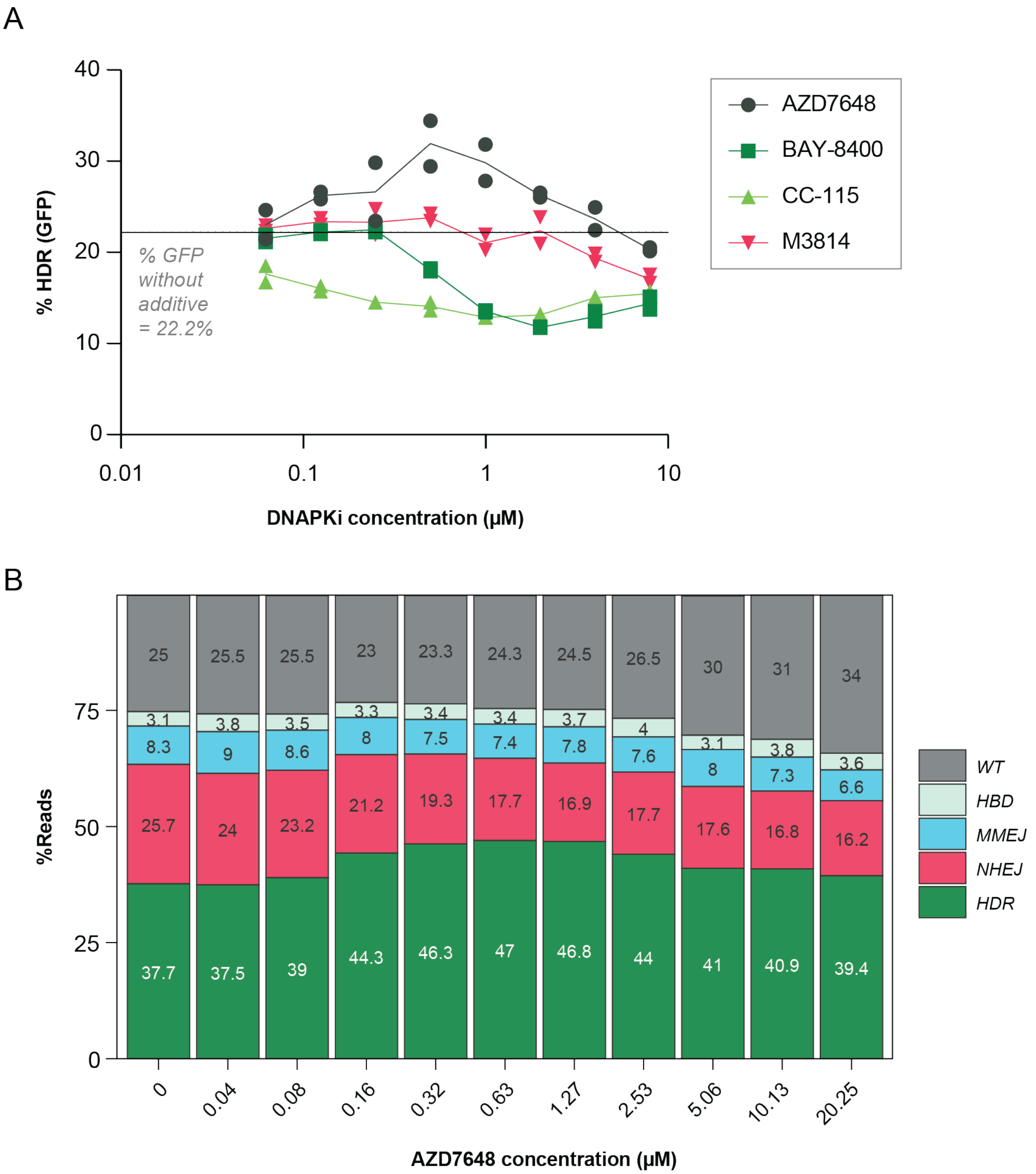

***Supplementary Figure 4.1*: DNAPKi dose response curves.** (A) Dose response curves when adding different DNAPKcs-targeting small molecules to an HSPC editing protocols using HBB-UbC-GFP AAV6 (MOI = 2500, *n* = 2, 2x10^6 cells/cuvette, split across 33 DNAPKi conditions). (B) Dose response curve of the effect of different types editing outcomes at *HBB* as determined by NGS when AZD7648 is added to HBB-SNP AAV6-containing media post nucleofection. (MOI = 2500, *n* = 3, 2.75x10^6 cells/cuvette, split across 11 AZD7648 concentrations, mean is depicted). (A-B) Source data are provided as a Source Data file.

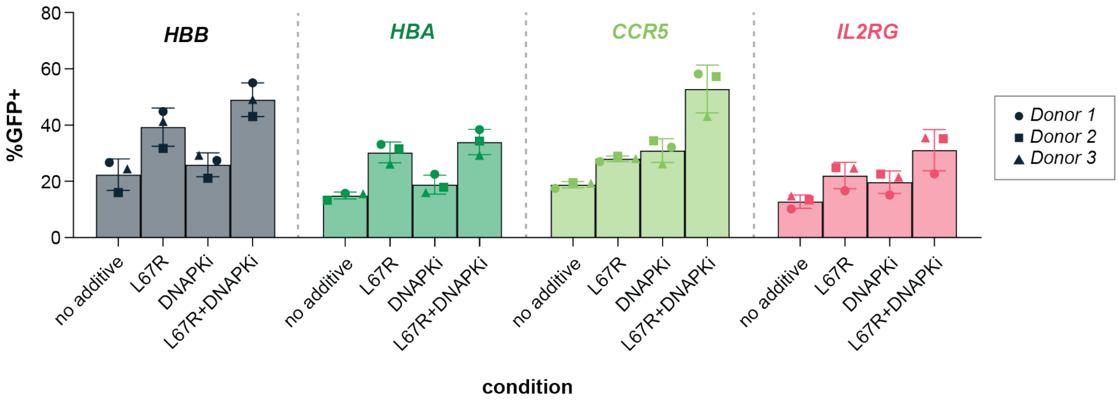

Supplementary Figure 4.2 **(related to Figure 4A): Editing at different clinically relevant loci using GFP-encoding AAV6, purified i53 variant protein, and AZD7648.** %GFP-expressing cells (%HDR) when L67R (0.8 mg/mL) is incorporated to an HSPC editing protocol for GFP knock-ins; post editing, cells were resuspended in media containing AAV6 targeted at *HBB*, *HBA*, *CCR5*, and *IL2RG* (MOI = 2500) with and without the addition of a DNAPKi (AZD7648, 0.5 µM). Cells were analyzed via flow cytometry 4 d post nucleofection; %GFP of live cells is shown. *n* = 3 independent HSPC donors (all male, $1.25x10^6$ cells/cuvette, split +/- DNAPKi) and mean +/- SD depicted. Source data are provided as a Source Data file.

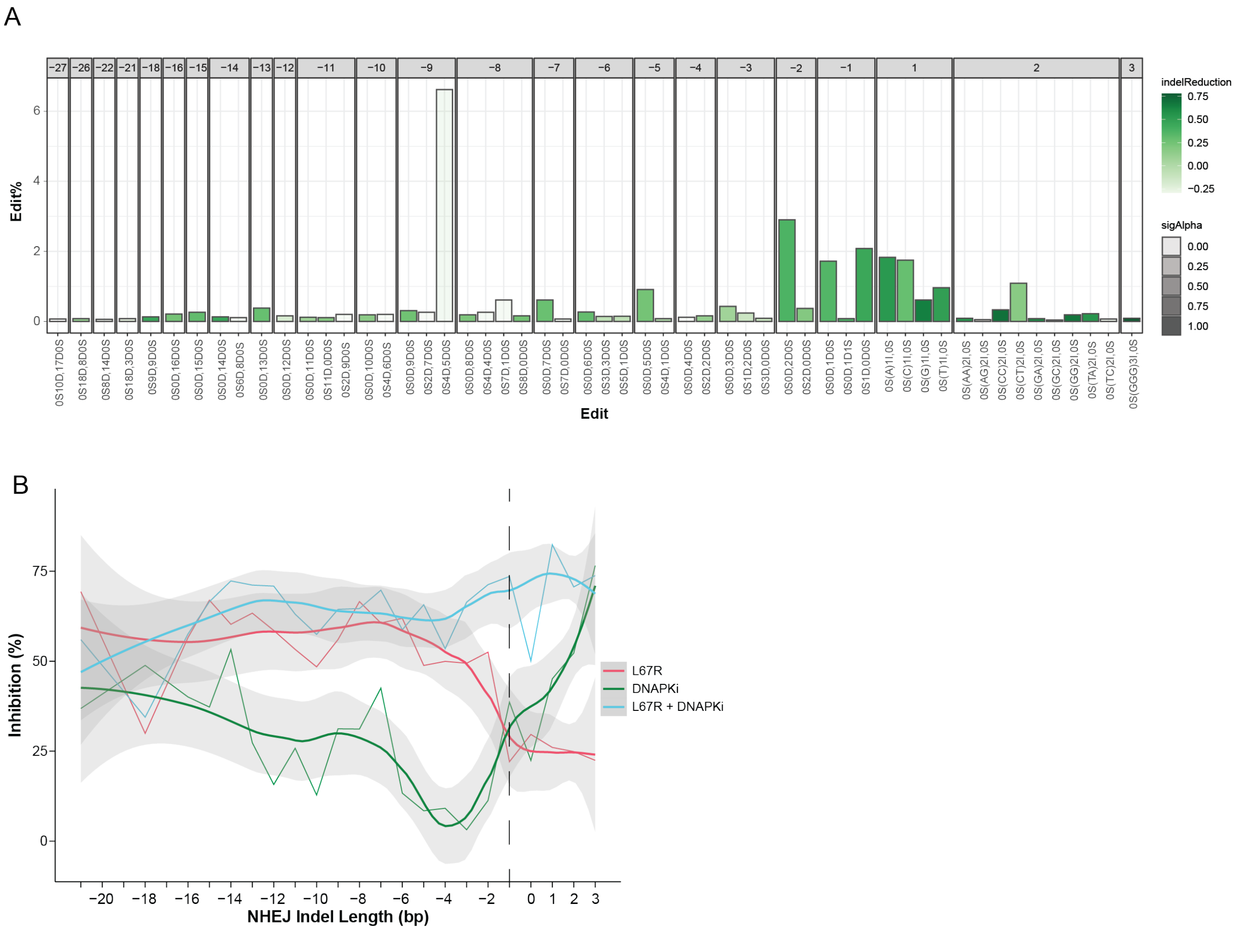

Supplementary Figure 4.3 **(related to Figure 4B): DNAPKi-sensitive INDELs and a comparison to 53BP1i-sensitive INDELs.** (A) Contribution of individual INDEL editing outcomes at *HBB* and sensitivity to DNAPKi AZD7648 (HDR/WT/HBD excluded, only indels with >0.1% reads shown). Bar shading represents reduction when DNAPKi small molecule is used during editing. White fill represents edits that did not result in a dose-dependent reduction of contribution when i53 variants were used. n = 3 different HSPC donors. An individual dose-response curve (four parameters, nonlinear) was fit for each variant and only those with significant association (p -< 0.01) were categorized as i53 variant responsive. (B) Differential effects of NHEJ inhibitors (L67R and AZD7648) alone and in combination on the %inhibition individual NHEJ-derived INDELs at *HBB* (MMEJ excluded), at different indel lengths. A vertical dashed line indicates the indel length at which DNAPKi small molecule yields better inhibition than 53BP1 inhibitor L67R. n = 3 different HSPC donors. (A-B) Source data are provided as a Source Data file.

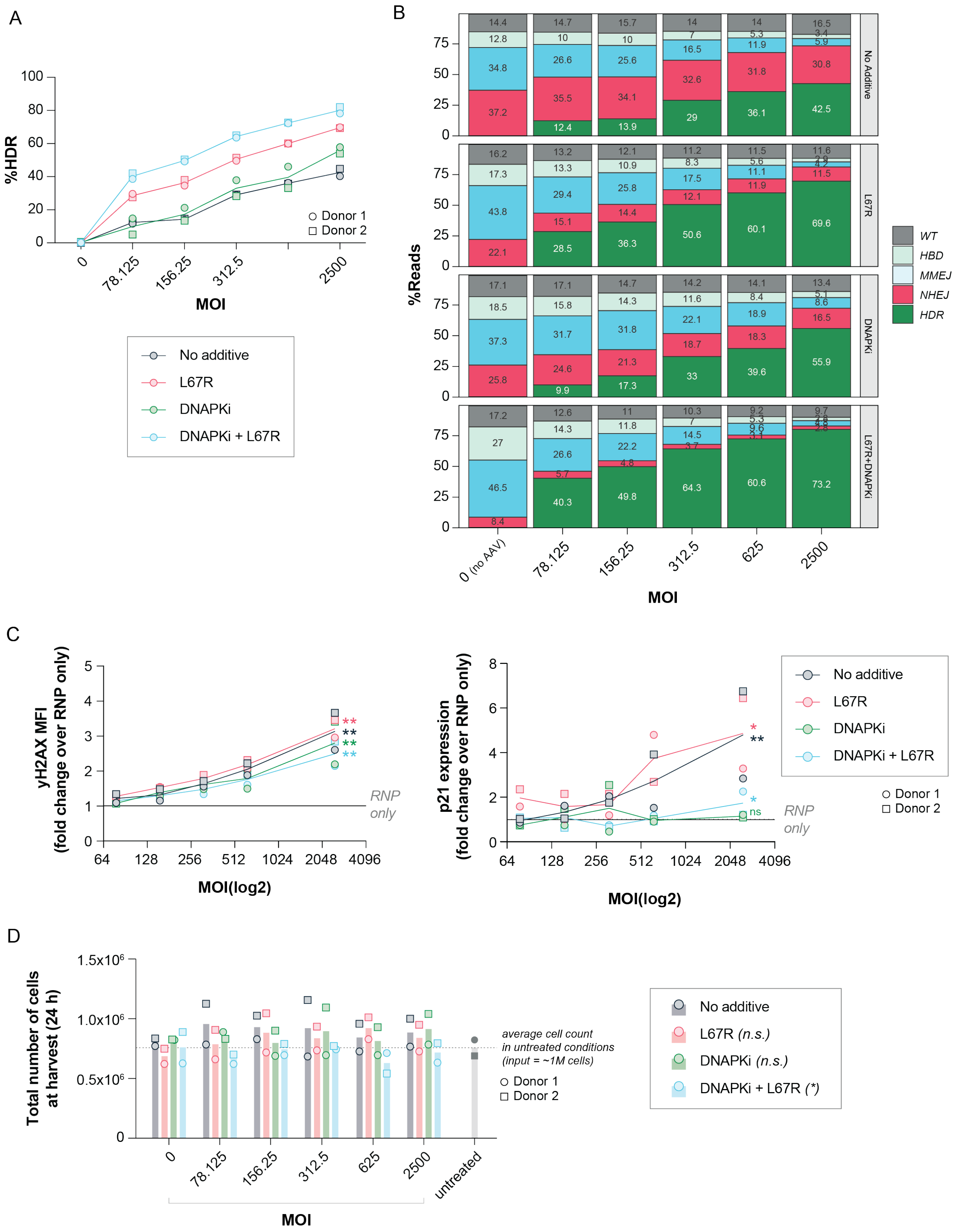
A
%HDR
100
80
60
40
20
0
0
78.125
156.25
312.5
2500
MOI
○ Donor 1
□ Donor 2
No additive
L67R
DNAPKi
DNAPKi + L67R
B
%Reads
75
50
25
0
No Additive
L67R
DNAPKi
L67R+DNAPKi
14.4
12.8
34.8
37.2
14.7
10
26.6
35.5
12.4
15.7
10
25.6
34.1
13.9
14
7
16.5
32.6
29
14
5.3
11.9
31.8
36.1
16.5
3.4
5.9
30.8
42.5
16.2
17.3
43.8
22.1
13.2
13.3
29.4
15.1
28.5
12.1
10.9
25.8
14.4
36.3
11.2
8.3
17.5
12.1
50.6
11.5
5.6
11.1
11.9
60.1
11.6
11.5
69.6
17.1
18.5
37.3
25.8
17.1
15.8
31.7
24.6
9.9
14.7
14.3
31.8
21.3
17.3
14.2
11.6
22.1
18.7
33
14.1
8.4
18.9
18.3
39.6
13.4
5.1
8.6
16.5
55.9
17.2
27
46.5
8.4
12.6
14.3
26.6
5.7
40.3
11
11.8
22.2
4.8
49.8
10.3
7
14.5
3.7
64.3
9.2
5.3
9.6
60.6
9.7
73.2
0 (no AAV)
78.125
156.25
312.5
625
2500
MOI
WT
HBD
MMEJ
NHEJ
HDR
C
yH2AX MFI
(fold change over RNP only)
5
4
3
2
1
0
64
128
256
512
1024
2048
4096
MOI(log2)
**
**
**
**
RNP
only
p21 expression
(fold change over RNP only)
8
6
4
2
0
64
128
256
512
1024
2048
4096
MOI(log2)
*
**
*
ns
RNP
only
No additive
L67R
DNAPKi
DNAPKi + L67R
○ Donor 1
□ Donor 2
D
Total number of cells
at harvest (24 h)
$1.5x10^6$
$1.0x10^6$
$0.5x10^6$
0
0
78.125
156.25
312.5
625
2500
untreated
MOI
average cell count
in untreated conditions
(input = ~1M cells)
○ Donor 1
□ Donor 2
No additive
L67R (n.s.)
DNAPKi (n.s.)
DNAPKi + L67R (*)

***Supplementary Figure 4.4* (related to Figure 4C): MOI titration of the HBB-SNP AAV6 donor with L67R and AZD7648.** (A) %HDR in cells treated with no additive, L67R (0.8 mg/mL), AZD7648 (0.5 μM), or both L67R and AZD7648 (n = 2 CD34+ HSPC donors, 1.2x10^7 cells/cuvette, split across 6 MOI conditions +/- DNAPKi). (B) Impact of MOI on the different types of editing outcomes as determined by NGS for both no additive and L67R only conditions. Stacked bars represent the contribution for different repair outcome categories. n = 2 separate HSPC donors and means are depicted. (C) Induction of the DNA Damage Response (DDR) as measured by expression of P21 and phosphorylation of histone H2AX (yH2AX) 24 hours post nucleofection and the differential effects for each editing condition across MOIs. n = 3 different HSPC donors. Significance is provided by slope of linear regression being different from zero. n.s.: not significant, *p < 0.05; **p < 0.01. (D) Total cells counted at harvest (24 h post nucleofection) for each editing condition tested (untreated condition cells counts are shown in grey, the average of which is depicted as a dotted line).– The only additive condition that impacted recovery significantly was the combination of DNAPKi and the i53 variant n = 2 separate HSPC donors and mean is depicted for each condition. n.s.: not significant, *p < 0.05. Analysis by two-way ANOVA with post-hoc comparison of the main effect of additive. (A-E) Source data are provided as a Source Data file.

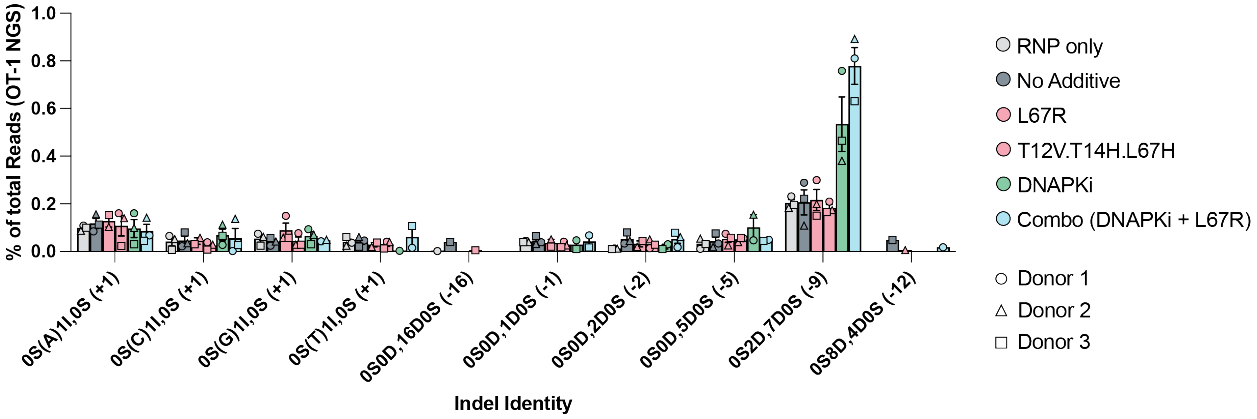

***Supplementary Figure 4.5* (related to Figure 4D): profile of indels in the OT-1 off target site when using the gRNA targeting the *HBB* locus.** The most prevalent edit is a 9 nucleotide deletion, which increases 2-3 fold when using DNAPKi small molecule. *n* = 3 separate donors and mean +/- SEM are depicted. Source data are provided as a Source Data file.

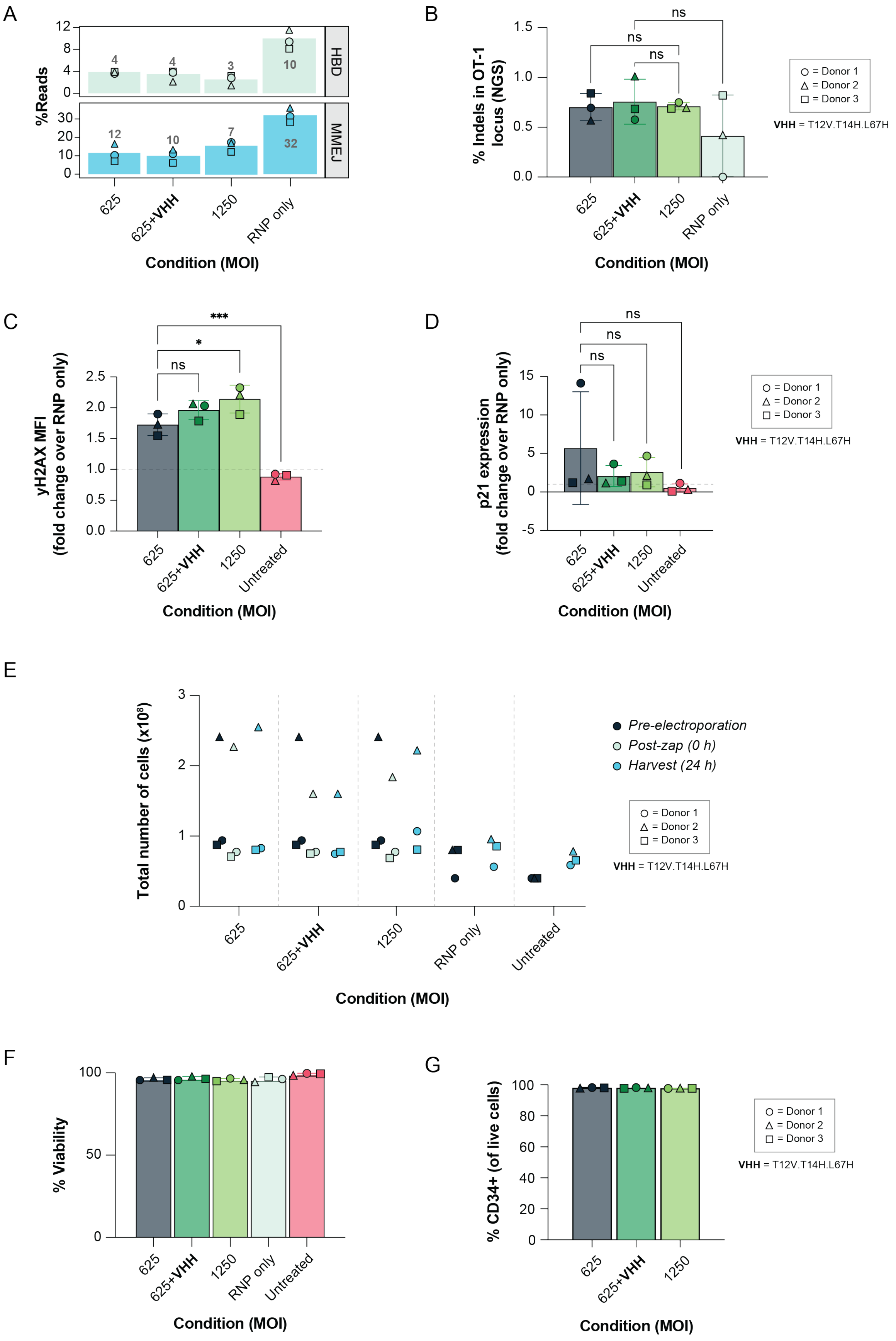

A
%Reads
12
8
4
0
30
20
10
0
HBD
MMEJ
4
4
3
10
12
10
7
32
625
625+VHH
1250
RNP only
Condition (MOI)
B
% Indels in OT-1 locus (NGS)
1.5
1.0
0.5
0.0
ns
ns
ns
625
625+VHH
1250
RNP only
Condition (MOI)
○ = Donor 1
△ = Donor 2
□ = Donor 3
VHH = T12V.T14H.L67H
C
yH2AX MFI (fold change over RNP only)
2.5
2.0
1.5
1.0
0.5
0.0
***
*
ns
625
625+VHH
1250
Untreated
Condition (MOI)
D
p21 expression (fold change over RNP only)
15
10
5
0
-5
ns
ns
ns
625
625+VHH
1250
Untreated
Condition (MOI)
○ = Donor 1
△ = Donor 2
□ = Donor 3
VHH = T12V.T14H.L67H
E
Total number of cells (x10^8)
3
2
1
0
625
625+VHH
1250
RNP only
Untreated
Condition (MOI)
Pre-electroporation
Post-zap (0 h)
Harvest (24 h)
○ = Donor 1
△ = Donor 2
□ = Donor 3
VHH = T12V.T14H.L67H
F
% Viability
100
50
0
625
625+VHH
1250
RNP only
Untreated
Condition (MOI)
G
% CD34+ (of live cells)
100
80
60
40
20
0
625
625+VHH
1250
Condition (MOI)
○ = Donor 1
△ = Donor 2
□ = Donor 3
VHH = T12V.T14H.L67H

***Supplementary Figure 5.1* (Related to Figure 5A): Medium scale production run editing outcomes and cell health metrics.** (A) HBD and MMEJ editing outcomes in CD34+ HSPCs from 3 donors that were edited with Cas9 RNP and HBB-SNP AAV6 at medium scale (~200 M HSPCs per condition for each donor). *n* = 3 different HSPC donors. (B) %INDELs at known off-target site OT-1. *n* = 3 different HSPC donors and mean +/- SD depicted. Analysis by one-way ANOVA with post-hoc pairwise comparisons and multiple testing adjustment. n.s. = not significant. (C-D) Induction of DNA Damage Response (DDR) was measured by (C) yH2AX phosphorylation and (D) P21 expression 24 hours post nucleofection. (E) Total number of cells for each condition and donor pre-electroporation, post-electroporation (0 h), and at harvest (24 h). Cell numbers are also shown in Supplementary Table S5.3. (F) Percent viability of cells for each condition at harvest (24 h). and (G) %CD34+ of cells post cryopreservation. For panels C, D, F and G, *n = 3* different HSPC donors and mean +/- SD depicted. Analysis by one-way ANOVA with post-hoc pairwise comparisons and multiple testing adjustment. n.s. = not significant; *$p < 0.05$; ***$p < 0.001$. (A-D, G) Source data are provided as a Source Data file.

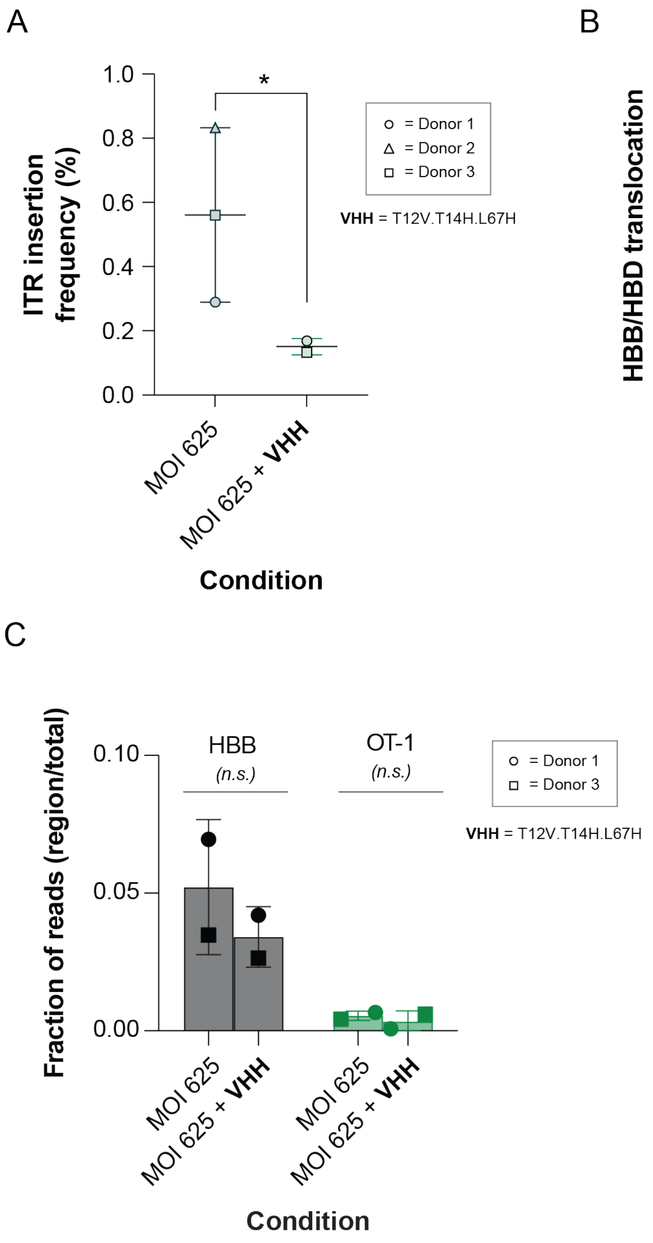

***Supplementary Figure 5.2*: Translocation-sequencing and Guide-Seq results**. (A) Summed frequency of AAV ITR sequence insertions identified at *HBB* and OT1 by translocation sequencing in bulk edited HSPC cells from medium scale production runs (MOI 625 and MOI 625 + T12V.T14H.L67H conditions only). *n = 3* separate donors and mean +/- SD depicted. Note that ITR integration was not identified in donor 2 using the MOI 625 + VHH editing condition. *p* = 0.0482 by two-sided unpaired t-test, setting missing conditions as 0. (B) Summed frequency of translocations identified between the *HBB* and *HBD* loci by translocation sequencing in bulk edited HSPC cells from medium scale production runs (MOI 625 and MOI 625 + T12V.T14H.L67H conditions only). *n = 3* separate donors and mean +/- SD depicted. Not statistically significant by two-sided unpaired t-test, setting missing conditions as 0. (C) Guide-seq characterization of cut sites for *HBB* locus targeting gene editing, with and without i53 variants used as additives. All the identified cutsites around *HBB* cutsite (on-target) and OT-1 (off-target) were aggregated into single columns. These were the only significant insertion sites not present in the untreated samples. *n* = 2 separate donors. All comparisons are non-significant by two-tailed paired t-test with multiple comparisons adjustment. (A-C) Source data are provided as a Source Data file.

***Supplementary Table S5.1*: Karyotyping of HSPC under different conditions**. In all conditions the evaluation was of normal karyotype, with some non-clonal, low level chromosomal gain/loss which was deemed normal for cell culture. *n* = 3 separate HSPC donors for all conditions; 100 spreads/condition.

Donor	Treatment	Total cells	Case notes	Number of Normal karyotype cells (46,XY/46,XX)	Chromosome gain/loss	Chr11 gain/loss (HBB)	Chr9 gain/loss (OT-1)
1	Untreated	100	Normal Karyotype	90	10	0	0
1	MOI 625	100	Normal Karyotype	82	18	0	1
1	MOI 625 + VHH	100	Normal Karyotype	80	20	1	1
2	Untreated	100	Normal Karyotype	89	11	0	1
2	MOI 625	100	Normal Karyotype	93	7	3	0
2	MOI 625 + VHH	100	Normal Karyotype	96	4	0	1
3	Untreated	100	Normal Karyotype	91	9	0	1
3	MOI 625	100	Normal Karyotype	88	12	1	0
3	MOI 625 + VHH	100	Normal Karyotype	84	16	1	1

***Supplementary Table S5.2*: Guide-seq characterization of cut sites for *HBB* locus targeting gene editing, with and without i53 variants used as additives.** This list includes all the matched integration records, whose left and right break points (BPs) have >= 1 UMI reads.

**Sample ID**	**L_BP_chr**	**L_Peak_Pos**	**R_Peak_Pos**	**Distance**	**Int_Dir**	**Region_reads**	**Region/Total(E-6)**	**Comment**	**Gene**
donor 1	chr11	5226983	5226984	0	+	25018	65824.37	On-Target	*HBB*
donor 1	chr9	101833600	101833601	0	+	2523	6638.22	OT-1	NA
donor 1	chr11	5226979	5226983	3	-	1397	3675.62	On-Target	*HBB*
donor 1 + VHH	chr11	5226983	5226984	0	+	23827	35629.74	On-Target	*HBB*
donor 1 + VHH	chr9	101833600	101833604	3	+	535	800.01	OT-1	NA
donor 1 + VHH	chr11	5226985	5226985	-1	-	4230	6325.34	On-Target	*HBB*
donor 3	chr11	5226983	5226984	0	+	16724	27364.18	On-Target	*HBB*
donor 3	chr9	101833600	101833603	2	+	2619	4285.27	OT-1	NA
donor 3	chr11	5226979	5226985	5	-	4562	7464.44	On-Target	*HBB*
donor 3 + VHH	chr9	101833600	101833601	0	-	3736	6112.93	OT-1	NA
donor 3 + VHH	chr11	5226983	5226984	0	+	25119	24988.98	On-Target	*HBB*
donor 3 + VHH	chr11	5226984	5226985	0	-	1415	1407.68	On-Target	*HBB*

Supplementary Table S5.3*: *Cell counts for medium scale production runs.

Donor	Treatment	Cells per condition pre-EP[a])	Total cells post-EP[a] (0 h)	Viability post-EP[a] (0 h)	Total cells at harvest (24 h)	Viability at harvest (24 h)
1	MOI 625	9.39E+07	7.75E+07	98.2%	8.28E+07	95.6%
1	MOI 625 + VHH	9.39E+07	7.75E+07	98.2%	7.49E+07	95.6%
1	MOI 1250	9.39E+07	7.75E+07	98.2%	1.07E+08	96.7%
1	RNP only	4.00E+07	NC[b]	NC[b]	5.64E+07	96.3%
1	Untreated	4.00E+07	NC[b]	NC[b]	5.86E+07	99.7%
2	MOI 625	2.41E+08	2.27E+08	97.1%	2.55E+08	97.2%
2	MOI 625 + VHH	2.41E+08	1.60E+08	97.7%	1.60E+08	97.9%
2	MOI 1250	2.41E+08	6.88E+07	96.7%	2.22E+08	95.8%
2	RNP only	8.00E+07	NC[b]	NC[b]	9.55E+07	94.5%
2	Untreated	4.00E+07	NC[b]	NC[b]	7.84E+07	98.5%
3	MOI 625	8.77E+07	7.08E+07	97.8%	8.03E+07	95.7%
3	MOI 625 + VHH	8.77E+07	7.50E+07	97.7%	7.75E+07	96.4%
3	MOI 1250	8.77E+07	1.84E+08	96.5%	8.08E+07	95.0%
3	RNP only	8.00E+07	NC[b]	NC[b]	8.55E+07	97.4%
3	Untreated	4.00E+07	NC[b]	NC[b]	6.56E+07	99.4%

[a] EP = electroporation
[b] NC = not counted

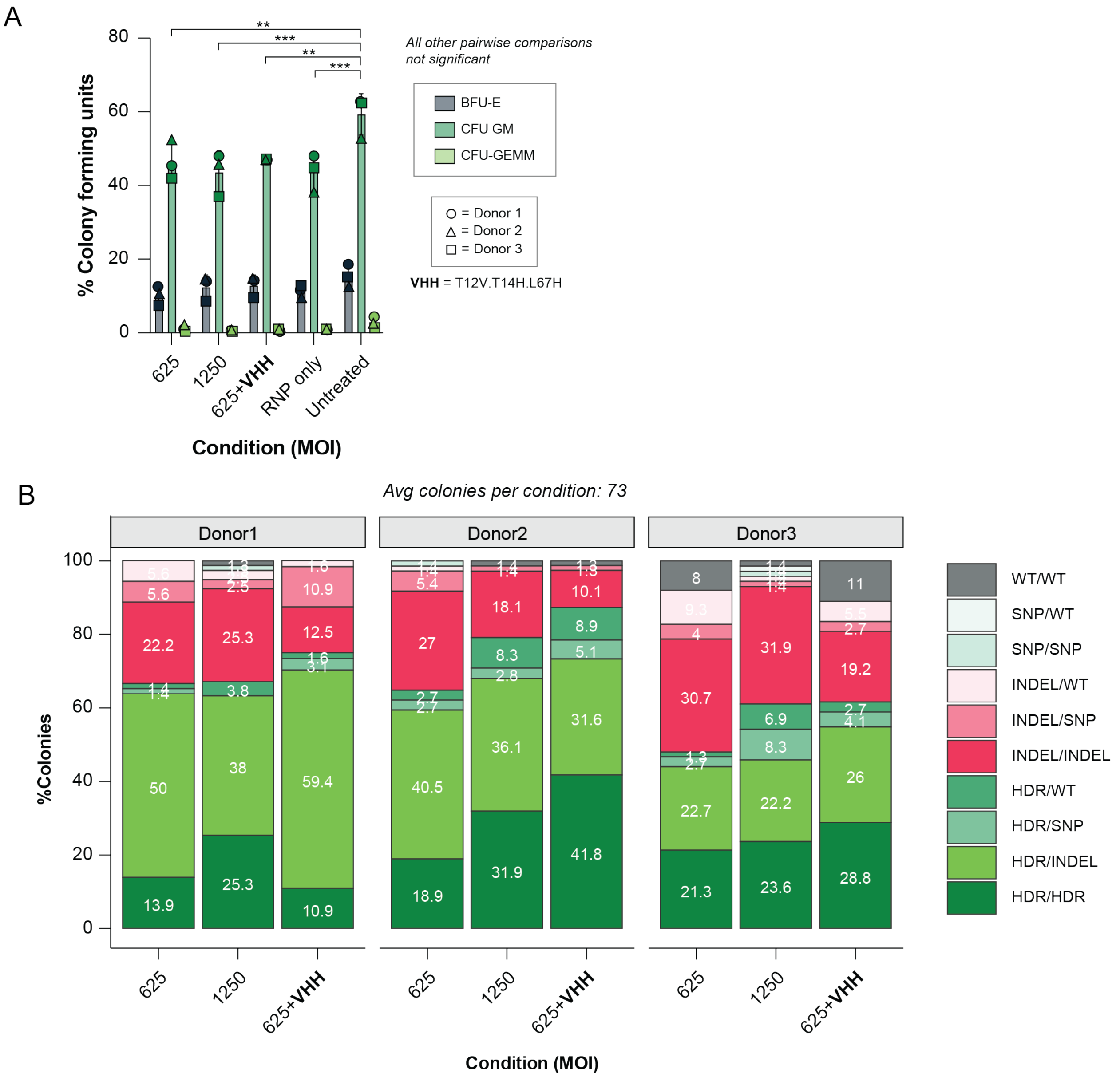

***Supplementary Figure 5.3* (related to Figure 5B): CFU counts and CFU sequencing results for medium scale production run editing.** (A) HSPC health and repopulation capacity was assessed by colony forming unit (CFU) recovery. CFUs were counted and stratified by type using a STEMvision colony counter instrument (StemCell Technologies): BFU-E (burst forming unit-erythroid), GM (granulocyte-macrophage progenitor), GEMM (multipotent progenitor granulocyte, erythrocyte, monocyte, megakaryocyte). *n* = 3 different HSPC donors and mean +/- SD depicted. Analysis by two-way ANOVA with post-hoc pairwise correction (Tukey correction). $^{**}p < 0.01$; $^{***}p < 0.001$. Only showing condition effect. (B) Breakdown of genotypes from the sequencing of individual colonies from each condition and donor. VHH = T12V.T14H.L67H (0.8 mg/mL). (A-B) Source data are provided as a Source Data file.

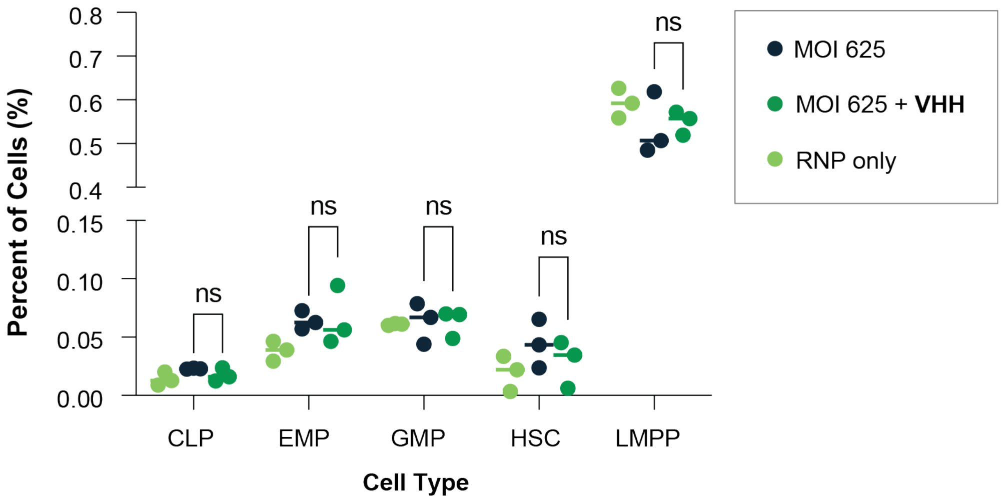

***Supplementary Figure 5.4*: i53 variant addition does not alter the cell mixture composition.** Single cell RNA sequencing (scRNA-seq) characterization of cell type composition from cell pools, 5 days post gene editing. For simplicity, only cell types that are relevant to engraftment (HSC and lineage-committed progenitors) are shown. Data was analyzed using separate t-test (two-tailed, unpaired) and adjusting for multiple comparisons. n.s.: $p > 0.1$. Cell type acronyms: CLP: common lymphoid progenitor; EMP: erythroid-megakaryocyte progenitor; GMP: granulocyte-monocyte progenitor; HSC: hematopoietic stem cell; LMPP: lymphoid-primed multipotential progenitor. VHH = T12V.T14H.L67H (0.8 mg/mL). Cell counts for all cell types can be found in Supplementary Table S5.2. Source data are provided as a Source Data file.

***Supplementary Table S5.4*: scRNA-Seq cell numbers.** VHH = T12V.T14H.L67H (0.8 mg/mL).

Cell type	MOI 625			MOI 625 + VHH			RNP only (no AAV)			Untreated		
	D1	D2	D3	D1	D2	D3	D1	D2	D3	D1	D2	D3
ASDC	0	0	1	0	2	4	0	3	1	0	0	0
BaEoMa	39	58	150	65	83	99	21	139	105	16	20	54
CD14 Mono	1	0	9	2	0	0	0	0	1	5	6	35
CD16 Mono	0	0	0	0	0	0	0	0	0	6	1	6
CD4 Effector	0	0	0	0	0	0	0	0	0	0	1	0
CD4 Memory	1	0	2	1	0	0	0	5	1	3	7	167
CD4 Naive	1	0	7	1	10	19	25	5	0	222	264	355
CD8 Effector_1	0	0	0	0	0	0	0	0	0	1	0	1
CD8 Memory	0	0	0	0	0	0	0	0	0	0	1	0
cDC2	0	0	0	0	0	3	0	0	1	0	0	0
CLP	65	68	139	50	62	96	22	88	92	307	174	377
Early Eryth	376	590	1154	537	894	862	365	1419	906	164	169	289
EMP	163	219	369	381	179	228	72	267	213	759	1062	1182
GMP	125	237	395	198	268	284	151	419	276	33	36	87
HSC	186	71	256	183	24	140	54	23	154	2452	2303	2078
Late Eryth	0	0	1	0	0	0	1	0	0	10	4	2
LMPP	1764	1460	2998	2311	2156	2106	1530	4062	2566	818	1024	429
Macrophage	0	0	0	0	0	0	0	0	0	0	0	5
Memory B	5	2	5	2	1	7	0	1	6	30	2	29
Naive B	0	0	0	0	0	0	1	0	0	2	2	8
NK	0	0	0	0	0	0	0	0	0	2	3	0
pDC	1	5	7	1	1	0	0	11	1	1	2	0
Plasma	42	59	97	79	60	25	21	54	40	120	63	106
Platelet	0	0	0	0	0	0	0	0	0	4	0	51
pre B	4	1	4	7	2	3	6	0	0	74	15	32
pre-mDC	5	28	65	16	26	49	1	50	45	0	0	1
pre-pDC	0	7	32	4	14	43	2	27	22	0	7	4
pro B	3	3	11	17	5	15	14	16	27	93	30	23
Prog Mk	71	202	207	184	82	73	152	262	137	12	16	22
transitional B	0	0	1	3	0	0	3	3	0	11	3	1
**Total**	2852	3010	5910	4042	3869	4056	2441	6854	4594	5145	5215	5344

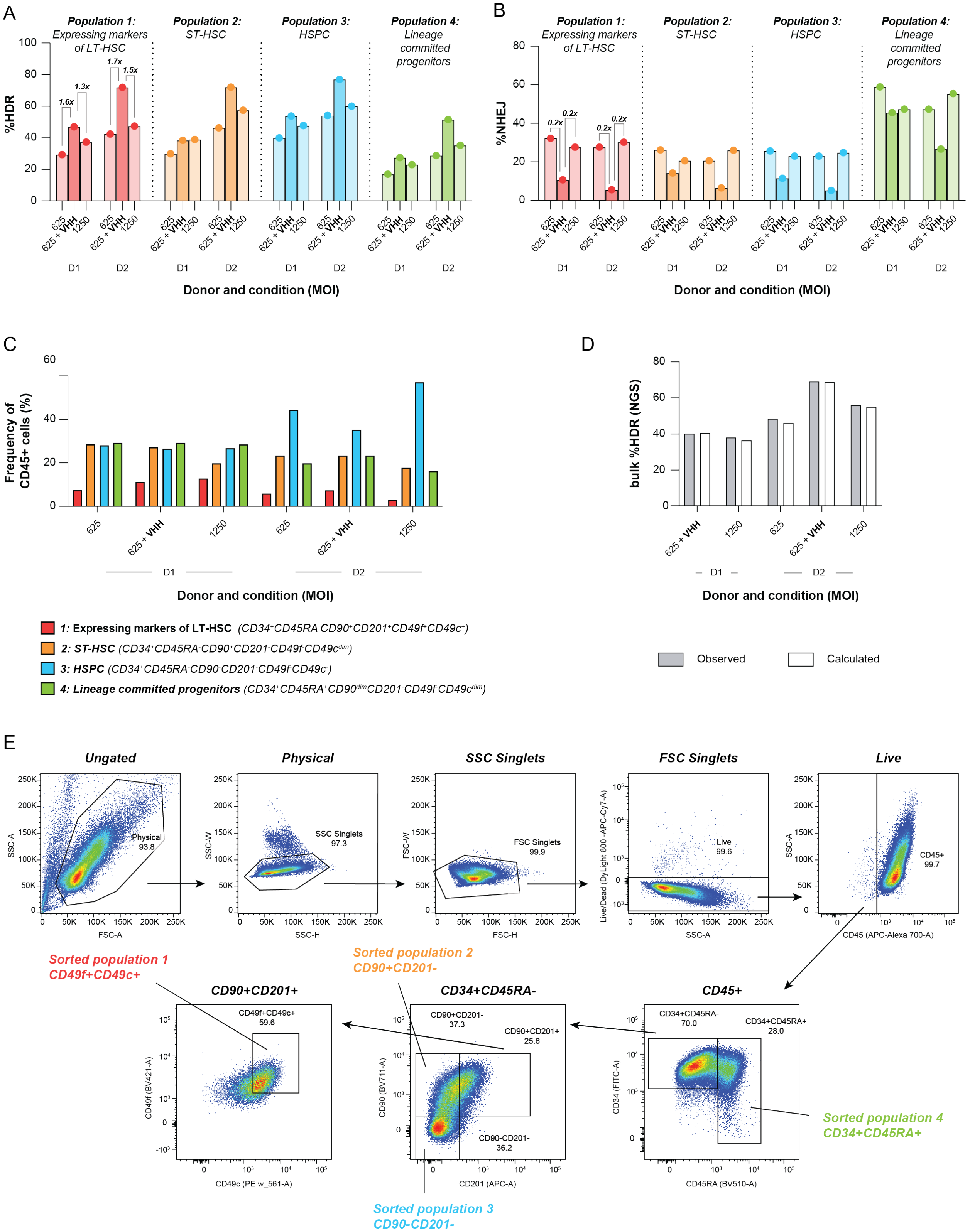
A
Population 1:
Expressing markers of LT-HSC
Population 2:
ST-HSC
Population 3:
HSPC
Population 4:
Lineage committed progenitors
%HDR
1.6x
1.3x
1.7x
1.5x
625
625 + VHH
1250
D1
D2
Donor and condition (MOI)
B
%NHEJ
0.2x
C
Frequency of CD45+ cells (%)
D
bulk %HDR (NGS)
1: Expressing markers of LT-HSC (CD34+CD45RA-CD90+CD201+CD49f+CD49c+)
2: ST-HSC (CD34+CD45RA-CD90+CD201-CD49f-CD49c^dim)
3: HSPC (CD34+CD45RA-CD90-CD201-CD49f-CD49c-)
4: Lineage committed progenitors (CD34+CD45RA+CD90^dim CD201-CD49f-CD49c^dim)
Observed
Calculated
E
Ungated
Physical
SSC Singlets
FSC Singlets
Live
Physical 93.8
SSC Singlets 97.3
FSC Singlets 99.9
Live 99.6
CD45+ 99.7
FSC-A
SSC-A
SSC-H
SSC-W
FSC-H
FSC-W
Live/Dead (DyLight 800 -APC-Cy7-A)
CD45 (APC-Alexa 700-A)
CD45+
CD34+CD45RA-
CD90+CD201+
CD34+CD45RA- 70.0
CD34+CD45RA+ 28.0
CD90+CD201- 37.3
CD90+CD201+ 25.6
CD90-CD201- 36.2
CD49f+CD49c+ 59.6
CD34 (FITC-A)
CD45RA (BV510-A)
CD90 (BV711-A)
CD201 (APC-A)
CD49f (BV421-A)
CD49c (PE w_561-A)
Sorted population 1
CD49f+CD49c+
Sorted population 2
CD90+CD201-
Sorted population 3
CD90-CD201-
Sorted population 4
CD34+CD45RA+

Supplementary Figure 5.5 **(related to Figure 5C): subpopulation sort and editing outcomes for HSPCs expressing markers associated with LT-HSCs, in medium-scale production runs.** (A) %HDR and (B) %NHEJ in HSPC subpopulations sorted from bulk edited cells as in Fig S4.2 (donor 1 and 2 only). VHH = T12V.T14H.L67H (0.8 mg/mL). D1 = donor 1. D2 = donor 2. (C) Observed frequency of sorted subpopulations for each donor and condition. (D) Calculated (from %HDR and frequency of each subpopulation) and observed %HDR in bulk edited cells. (E) Representative flow cytometry plots showing the sort gating strategy to isolate Single-Live populations of HSCs using designated markers. LT-HSC* (population 1): cells expressing markers associated with Long Term HSCs ($CD34^{+}CD45RA^{-}CD90^{+}CD201^{+}CD49f^{+}CD49c^{+}$); ST-HSC (population 2): 'Short-term' HSCs ($CD34^{+}CD45RA^{-}CD90^{+}CD201^{-}CD49f^{-}CD49c^{dim}$); HSPC (population 3): general HSPCs ($CD34^{+}CD45RA^{-}CD90^{-}CD201^{-}CD49f^{-}CD49c^{-}$); Lineage committed progenitors (population 4) ($CD34^{+}CD45RA^{+}CD90^{dim}CD201^{-}CD49f^{-}CD49c^{dim}$). (A-D) Source data are provided as a Source Data file.
