# Supplementary material for: Functional screening in human HSPCs identifies optimized protein-based enhancers of Homology Directed Repair
Source: Nat Commun. 2024 Mar 23;15:2625. doi: 10.1038/s41467-024-46816-5 (PMC10960832; doi:10.1038/s41467-024-46816-5)
Supplement: Supplementary file 3 — Description of Additional Supplementary Files [file 41467_2024_46816_MOESM3_ESM.pdf]

Description of Additional Supplementary Files

File Name: Supplementary Data 1
Description: Mass spectrometry data used for the additional validation of purified protein variants. GraphiteBio_3 = T12V.T14H.L67H, GraphiteBio_4 = 53BP1 Tudor domain, GraphiteBio_5 = T12Y.T14E.L67R, GraphiteBio_6 = i53.

File Name: Supplementary Data 2
Description: PDB validation reports for deposited structures.

File Name: Supplementary Data 3
Description: List of each unique indel, along with average % outcome contribution and average reduction when using i53 variants used in this study. For classification, MMEJ outcomes were designated empirically by POLQ gene knockdown (Supplementary figure S3.6), and every other outcome outside HDR/WT/HBD was categorized as NHEJ. INDELs are designated by the insertion ("I") or deletion ("D") length at either side of the cutsite (represented with a comma); e.g. 4D,5D represents a deletion of 4 nucleotides before cutsite and 5 nucleotides after cutsite.

File Name: Supplementary Data 4
Description: Description of the different sequencing outcomes associated with *HBD* recombination events, along with the % contribution to the total *HBD* fraction of results in amplicon sequencing analyses in this report. Outcomes are categorized by the mismatch (SNP) location in the template sequence. HBB template sequence:
ATGGTGCATCTGACTCCTGAGGAGAAGTCTGCCGTTACTGCCCTGTGGGGCAAGGTG
AACGTGGATGAAGTTGGTGGTGAGGCCCTGGGCAG; HBD template sequence:
ATGGTGCATCTGACTCCTGAGGAGAAGACTGCTGTCAATGCCCTGTGGGGCAAAGT
GAACGTGGATGCAGTTGGTGGTGAGGCCCTGGGCAG.

File Name: Supplementary Data 5
Description: DNA sequences for all plasmids used in this study (i53 variant expression lentiviral vector, AAV donor templates for HDR repair, and lentiviral vectors expressing shRNA for POLQ knockdown experiments).

File Name: Supplementary Data 6
Description: Tables referenced in the Methods section. Table M1: Standard cells numbers used in MaxCyte electroporation cuvettes; Table M2: Full list of parameters passed to Crispresso; Table M3: Antibodies and reagents for LT-HSC sort; Table M4: Trans-seq target list; Table M5: Trans-seq primer list.
