# Supplementary material for: Functional screening in human HSPCs identifies optimized protein-based enhancers of Homology Directed Repair
Source: Nat Commun. 2024 Mar 23;15:2625. doi: 10.1038/s41467-024-46816-5 (PMC10960832; doi:10.1038/s41467-024-46816-5)

Supplementary Data 1: Intact Mass Analysis by LC-MS

The data in this supplement was generated by JadeBio, Inc. and is reported here as is.

LC-MS without reduction and deglycosylation.

Summary of data

Tube Label	Sample Name	Intact Mass (Da)	
		Measured	Expected
3	GraphiteBio_3	8705.58	8550.79
4	GraphiteBio_4	13899.39	13896.58
5	GraphiteBio_5	8780.91	8762.01
6	GraphiteBio_6	8349.28; 8532.20	8646.99

Measured Intact Mass for GraphiteBio_3

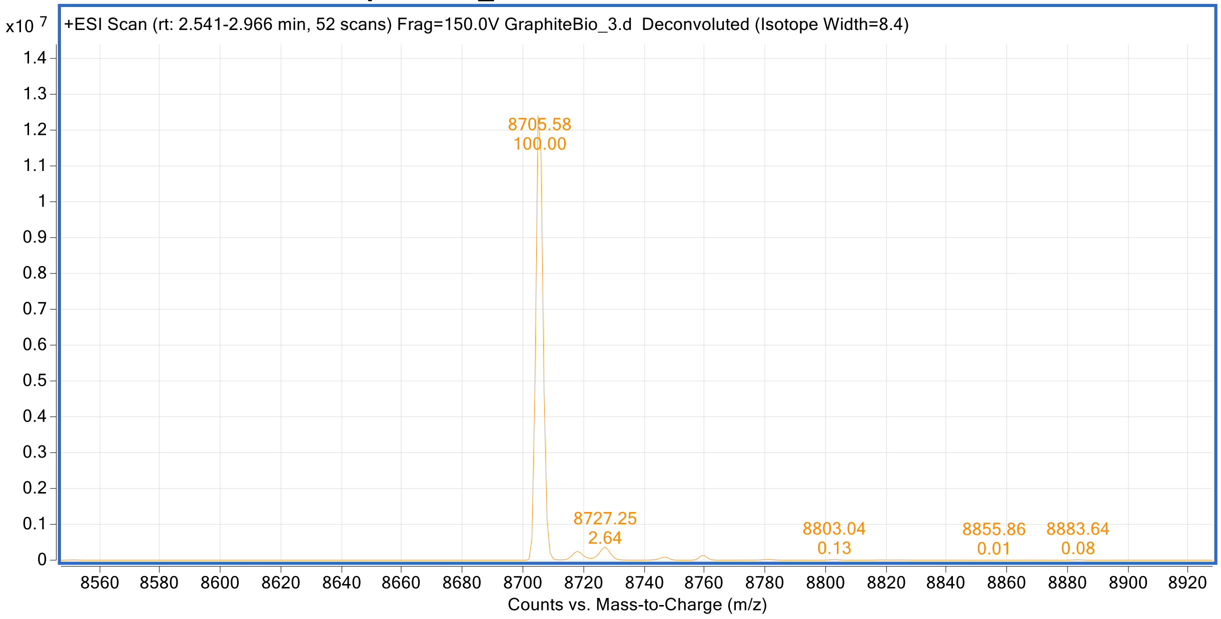

Measured Intact Mass for GraphiteBio_4

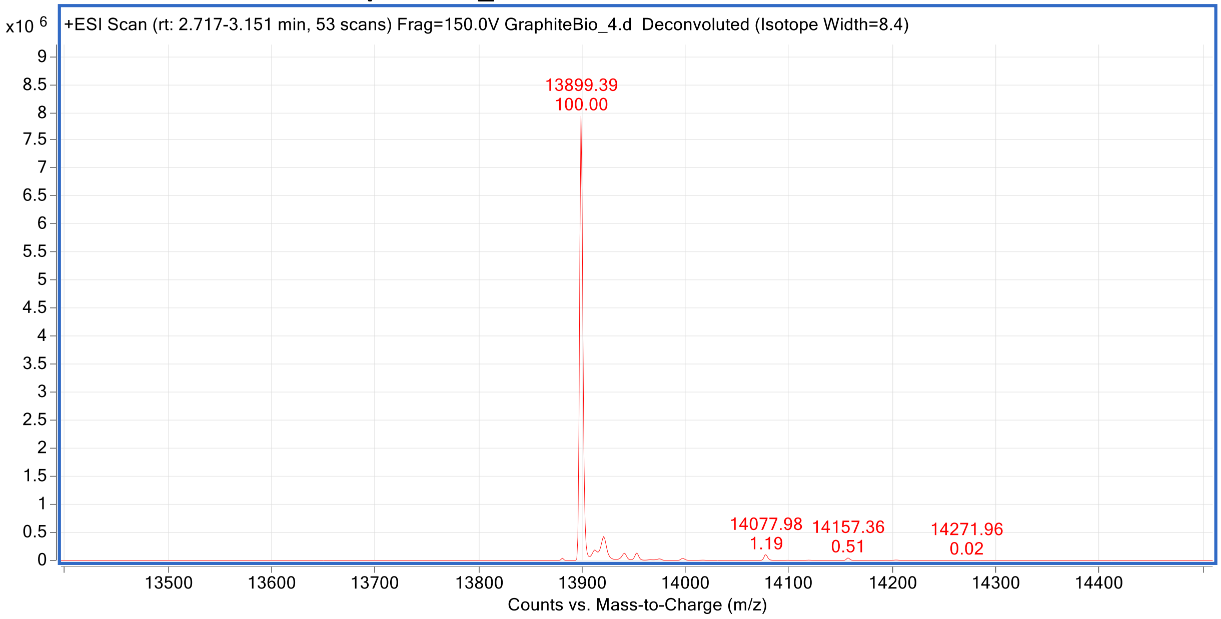

Measured Intact Mass for GraphiteBio_5

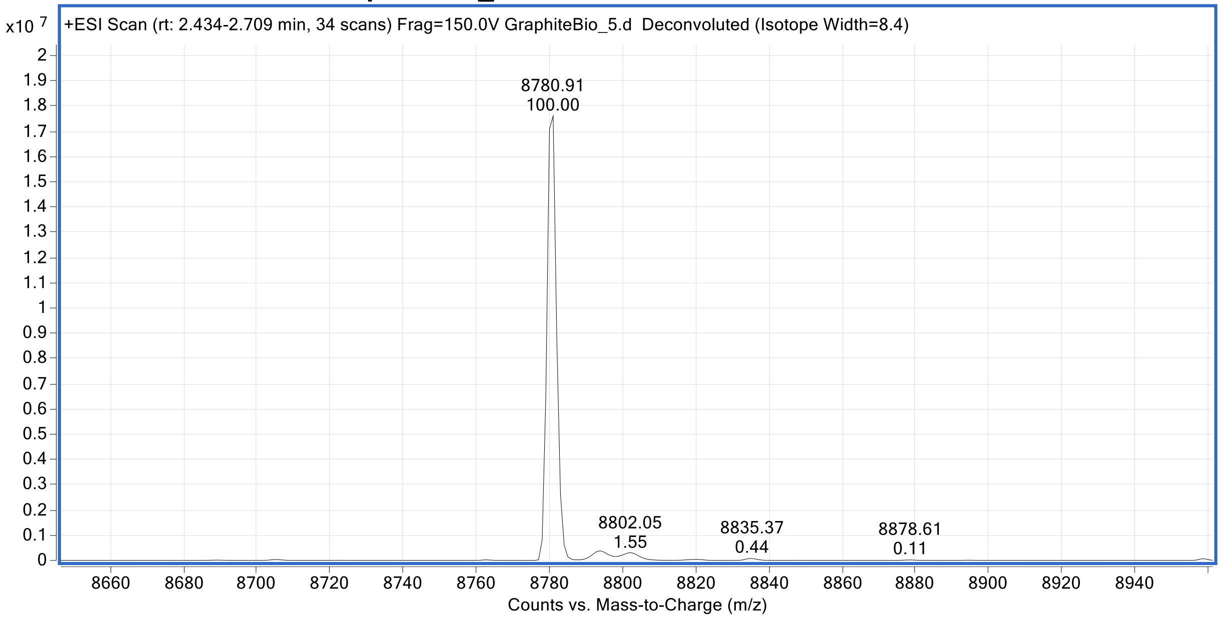

Measured Intact Mass for GraphiteBio_6

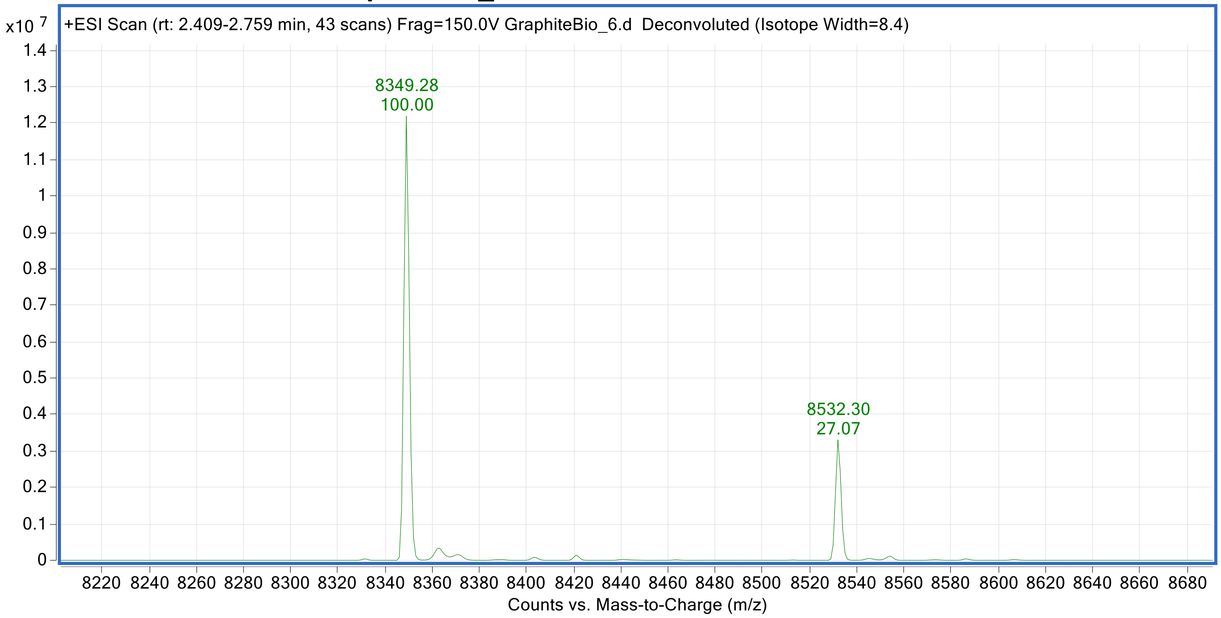

Supplement: Supplementary file 4 — Supplementary Data 1 [file 41467_2024_46816_MOESM4_ESM.pdf]
