# Supplementary material for: Functional screening in human HSPCs identifies optimized protein-based enhancers of Homology Directed Repair
Source: Nat Commun. 2024 Mar 23;15:2625. doi: 10.1038/s41467-024-46816-5 (PMC10960832; doi:10.1038/s41467-024-46816-5)
Supplement: Supplementary file 10 — Reporting Summary [file 41467_2024_46816_MOESM10_ESM.pdf]

nature portfolio

Corresponding author(s): Kristen L. Seim, Ph.D.

Last updated by author(s): Feb 9, 2024

Reporting Summary

Nature Portfolio wishes to improve the reproducibility of the work that we publish. This form provides structure for consistency and transparency in reporting. For further information on Nature Portfolio policies, see our Editorial Policies and the Editorial Policy Checklist.

Statistics

For all statistical analyses, confirm that the following items are present in the figure legend, table legend, main text, or Methods section.

n/a	Confirmed	
☐	☒	The exact sample size (*n*) for each experimental group/condition, given as a discrete number and unit of measurement
☐	☒	A statement on whether measurements were taken from distinct samples or whether the same sample was measured repeatedly
☐	☒	The statistical test(s) used AND whether they are one- or two-sided<br>*Only common tests should be described solely by name; describe more complex techniques in the Methods section.*
☐	☒	A description of all covariates tested
☐	☒	A description of any assumptions or corrections, such as tests of normality and adjustment for multiple comparisons
☐	☒	A full description of the statistical parameters including central tendency (e.g. means) or other basic estimates (e.g. regression coefficient) AND variation (e.g. standard deviation) or associated estimates of uncertainty (e.g. confidence intervals)
☐	☒	For null hypothesis testing, the test statistic (e.g. *F*, *t*, *r*) with confidence intervals, effect sizes, degrees of freedom and *P* value noted<br>*Give P values as exact values whenever suitable.*
☒	☐	For Bayesian analysis, information on the choice of priors and Markov chain Monte Carlo settings
☒	☐	For hierarchical and complex designs, identification of the appropriate level for tests and full reporting of outcomes
☒	☐	Estimates of effect sizes (e.g. Cohen's *d*, Pearson's *r*), indicating how they were calculated

Our web collection on statistics for biologists contains articles on many of the points above.

Software and code

Policy information about availability of computer code

Data collection	No code was used to collect the data.
Data analysis	Processing of FASTQ files from amplicon sequencing was performed using CRISPResso2 (v2.1.0). Parameter information can be found in Methods section of text. Initial parsing of the CRISPResso2 reports to generate preprocessed data was performed using a simple custom Python script.<br>Guide-seq and Translocation-seq: raw reads were demultiplexed using bcl2fastq (v2.20.0.422). Reads were aligned using BWA v0.7.17-r1188. Remaining steps were performed using a proprietary pipeline by GeneGoCell (see methods section).<br>Analysis of screening libraries: custom code (using R v4.2.0) was used to parse, analyze and generate enrichment values for hits.<br>scRNA-seq: analyses were performed using R (v4.2.0), in particular Seurat (v.4.3.0) package and dittoSeq (v1.8.1) and ggplot2.<br>For all statistical analyses and some of the plots, GraphPad Prism (v 9.5.1) was used. Remaining data parsing (especially of CRISPResso preprocessed data, and screening data), reshaping and plotting was performed using R (v4.1.2 or v4.2.0, where indicated) on Rstudio (v1.3.1093). Packages used: ggplot2(v3.4.3) for plotting; drc(v3.0-1) for dose response generation.<br>Structure renders from crystallography data were built using COOT and refined using Refmac (see methods section). Visualizations were generated and annotated using PyMOL software (v2.0).<br>Flow cytometry data was analyzed using FlowJo 10 software.

For manuscripts utilizing custom algorithms or software that are central to the research but not yet described in published literature, software must be made available to editors and reviewers. We strongly encourage code deposition in a community repository (e.g. GitHub). See the Nature Portfolio guidelines for submitting code & software for further information.

Data

Policy information about availability of data

All manuscripts must include a data availability statement. This statement should provide the following information, where applicable:

- Accession codes, unique identifiers, or web links for publicly available datasets
- A description of any restrictions on data availability
- For clinical datasets or third party data, please ensure that the statement adheres to our policy

Atomic coordinates for crystal structures have been deposited in the Protein Data Bank (PDB) under accession codes: 8SVG, 8SVH, 8SVI, 8SVJ, and 8T2D. Sequencing data (Amplicon Sequencing for on- and off- target editing; scRNAseq) is deposited on GEO under the accession code GSE242757.

Mass Spectrometry raw data (used for additional validation of purified protein variants) is not available due to provider policy.

Amplicon NGS (on and off-target HBB and i53 variant enrichment) and scRNA-seq data has been deposited in NCBI's Gene Expression Omnibus45 and are accessible through GEO Series accession number GSE242757 (https://www.ncbi.nlm.nih.gov/geo/query/acc.cgi?acc=GSE242757).

The reference genome used alignments in sequencing-based assays was always GRCh38 (hg38; www.ncbi.nlm.nih.gov/datasets/genome/GCF_000001405.26/).

Research involving human participants, their data, or biological material

Policy information about studies with human participants or human data. See also policy information about sex, gender (identity/presentation), and sexual orientation and race, ethnicity and racism.

Reporting on sex and gender	Cell lines: HEK293T cells (and derived Lenti-X HEK293T cells) were purchased from vendor. They are originally derived from a female fetus. K562 cells were purchased from vendor and are originally derived from a female patient.<br><br>Primary hematopoietic (CD34+) cells used in this study were purchased from AllCells, having been originally extracted from multiple different donors (which were used as replicates in most of the experiments described in this manuscript). Sex information of donors was available but was not considered relevant for this study and it was not analyzed as a variate or covariate.<br>UPDATED: the sex (as XX or XY) of HSPC donors is disclosed only where relevant, in the experiments reporting IL2RG locus editing. All donors used in that regard were XY.
Reporting on race, ethnicity, or other socially relevant groupings	N/A<br><br>Cell lines: race and ethnicity information was not available from vendors.<br><br>Primary hematopoietic (CD34+) cells used in this study were purchased from AllCells, having been originally extracted from multiple different donors (which were used as replicates in most of the experiments described in this manuscript). Age, race and ethnicity information of donors was available but due to the lack of data supporting the influence of these variables on genome editing outcomes, these factors were not considered relevant for this study and it were not analyzed as a variate or covariate.
Population characteristics	N/A. Age, race and ethnicity information of donors was available but due to the lack of data supporting the influence of these variables on genome editing outcomes, these factors were not considered relevant for this study and it were not analyzed as a variate or covariate.
Recruitment	N/A. All the human materials used (leukopak and CD34+ HSPCs) were purchased from All Cells, a company specialized in the procurement of human-derived hematological tissues and services. Collection protocols and donor-informed consent are approved by an Institutional Review Board (IRB) by AlphaIRB (OHRP/FDA registration number IRB00006205). HIPAA compliance and approved protocols are also followed. Informed consent was obtained on every donor.
Ethics oversight	N/A. See 'Recruitment' section above for primary cell collection and donor recruitment oversight.

Note that full information on the approval of the study protocol must also be provided in the manuscript.

Field-specific reporting

Please select the one below that is the best fit for your research. If you are not sure, read the appropriate sections before making your selection.

☒ Life sciences ☐ Behavioural & social sciences ☐ Ecological, evolutionary & environmental sciences

For a reference copy of the document with all sections, see nature.com/documents/nr-reporting-summary-flat.pdf

Life sciences study design

All studies must disclose on these points even when the disclosure is negative.

Sample size	Sample size (biological replicates of editing experiments, mostly) was fixed to 3 for most experiments, unless otherwise mentioned (Fig 3 and

Sample size	Fig 5 have panels utilizing only 2 replicates due to resource intensive nature of experiments; Figure 3 and Figure 4 have panels where larger sample sizes were used, mostly combining different experiments).<br><br>Screening libraries were performed in 3 independent technical replicates (same cell line and procedure repeated 3 times).<br><br>No prior sample size calculations were performed, instead resorting to these numbers as conditioned by resource availability and intrinsic variability of the experiments.
Data exclusions	A pre-established exclusion criteria that removes samples where total editing % or Amplicon Sequencing reads is below a threshold was implemented. It impacted less than 5% of the datapoints from this manuscript, and repeat experiments were performed for those samples.
Replication	All experiments used biological replicates (cells coming from different human donors) where possible, and unless otherwise mentioned (e.g. when using cell lines). Replicates were performed in parallel (often by different operators) with some exceptions where data from separate experiments is combined (explicitly mentioned in the figure captions).
Randomization	Not applicable - same HSPC donor samples were used across conditions, with the variable being the cell treatment.
Blinding	Blinding was not possible to investigators performing editing experiments, due to their involvement in adding the reagents to the editing process. Downstream of this (sample processing, amplicon sequencing, data analysis) samples were blinded to investigators until the summary/plotting step, when labels were decoded.

Reporting for specific materials, systems and methods

We require information from authors about some types of materials, experimental systems and methods used in many studies. Here, indicate whether each material, system or method listed is relevant to your study. If you are not sure if a list item applies to your research, read the appropriate section before selecting a response.

Materials & experimental systems

n/a	Involved in the study
☐	☒ Antibodies
☐	☒ Eukaryotic cell lines
☒	☐ Palaeontology and archaeology
☒	☐ Animals and other organisms
☒	☐ Clinical data
☒	☐ Dual use research of concern
☒	☐ Plants

Methods

n/a	Involved in the study
☒	☐ ChIP-seq
☐	☒ Flow cytometry
☒	☐ MRI-based neuroimaging

Antibodies

Antibodies used	anti-H2A.X Phospho (Ser139)-PE (BioLegend, #613411, clone 2F3)<br>anti-p21 (CST, #2947; clone 12D1)<br>anti-alpha tubulin (Abcam ab4074; clone N/A)<br>anti-CD34-Alexa-488 (Biolegend, #343518; clone 581)<br>anti-CD38-PE-Cy7 (BD Biosciences, #560677; clone HIT2)<br>anti-CD45RA-BV510 (Biolegend, #304142; clone HI100)<br>anti-CD49c-PE (Biolegend, #343803; clone ASC-1)<br>anti-CD49f-BV421 (Biolegend, #313624; clone GoH3)<br>anti-CD90-BV711 (Biolegend, #328139; 5E10)<br>anti-CD201-APC (Biolegend, #351906, RCR-401)<br>anti-CD45-Alexa-700 (Biolegend, #304024; clone HI30)
Validation	No novel antibodies were used for this manuscript.<br><br>All the antibodies used in this study (except the Tubulin loading control, see at the bottom of the list) are monoclonal and have been verified and quality tested for flow cytometry by the vendor. In addition, the monoclonal antibody clones have been identified in human leukocyte differentiation antigen (HLDA) workshops, as follows:<br><br>anti-H2A.X Phospho (Ser139)-PE (BioLegend, #613411). Validated by provider by flow cytometry.<br>anti-p21 (CST, #2947). Validated by provider by knockout-WB.<br>anti-alpha tubulin (Abcam ab4074). Validated by provider with antibodies for same target (western blot).<br>anti-CD34-Alexa-488 (Biolegend, #343518; clone 581)<br>Workshop: VMA27<br>RRID: AB_1937203<br><br>anti-CD38-PE-Cy7 (BD Biosciences, #560677; clone HIT2)<br>Workshop: IIT155<br>RRID: AB_1727473

anti-CD45RA-BV510 (Biolegend, #304142; clone HI100)
Workshop: IV N906
RRID: AB_2561947

anti-CD49c-PE (Biolegend, #343803; clone ASC-1)
Workshop: VIA002
RRID: AB_1731941

anti-CD49f-BV421 (Biolegend, #313624; clone GoH3)
Workshop: IVP55
RRID: AB_2562244

anti-CD90-BV711 (Biolegend, #328139; 5E10)
Workshop: VM07, BP222: VI BP28, E046
RRID: AB_2734316

anti-CD45-Alexa-700 (Biolegend, #304024; clone HI30)
Workshop: IV N816
RRID: AB_493761

Monoclonal antibody for which no workshop was identified, but it was validated in literature:
anti-CD201-APC (Biolegend, #351906, RCR-401)
Reference : Chagraoui J, Girard S, Spinella JF, Simon L, Bonneil E, Mayotte N, MacRae T, Coulombe-Huntington J, Bertomeu T, Moison C, Tomellini E, Thibault P, Tyers M, Marinier A, Sauvageau G. UM171 Preserves Epigenetic Marks that Are Reduced in Ex Vivo Culture of Human HSCs via Potentiation of the CLR3-KBTBD4 Complex. Cell Stem Cell. 2021 Jan 7;28(1):48-62.e6. doi: 10.1016/j.stem.2020.12.002. PMID: 33417871.
Validation by flow cytometry
Workshop: N/A
RRID: AB_11126139

anti-H2A.X Phospho (Ser139)-PE (BioLegend, #613411, clone 2F3)
Reference: Falcone, M., Uribe, A.H., Papalazarou, V. et al. Sensitisation of cancer cells to radiotherapy by serine and glycine starvation. Br J Cancer 127, 1773–1786 (2022). https://doi.org/10.1038/s41416-022-01965-6
Validation by staining post-exposure of DNA damage inducing molecules.
Also validated by vendor for western blotting.

Anti-p21 (CST, #2947; clone 12D1)
Reference: Sheaff RJ, Singer JD, Swanger J, Smitherman M, Roberts JM, Clurman BE. Proteasomal turnover of p21Cip1 does not require p21Cip1 ubiquitination. Mol Cell. 2000 Feb;5(2):403-10. doi: 10.1016/s1097-2765(00)80435-9. PMID: 10882081.
Validation by western blot, immunoprecipitation, pulse-chase.
Additionally validated by provider by knockout western blot of HeLa lines.

anti-alpha tubulin (Abcam ab4074; clone N/A)
Polyclonal antibody.
Validated by vendor by immunostaining and by western blot of cell lysates with spike-in of recombinant alpha-tubulin.

Eukaryotic cell lines

Policy information about cell lines and Sex and Gender in Research

Cell line source(s)	Lenti-X HEK293T cells were purchased from Takara Bio and K562 cells were purchased from ATCC. CD34+ HSPCs were either purchased from AllCells (isolated from G-CSF-mobilized peripheral blood from healthy donors) or isolated from leuokopaks purchased from AllCells.
Authentication	Cell lines were not authenticated. CD34+ HSPCs were authenticated by provider.
Mycoplasma contamination	Routine mycoplasma testing was performed quarterly. Results were negative through the duration of this study.
Commonly misidentified lines (See ICLAC register)	No commonly misidentified cell lines were used.

Flow Cytometry

Plots

Confirm that:

☒ The axis labels state the marker and fluorochrome used (e.g. CD4-FITC).

☒ The axis scales are clearly visible. Include numbers along axes only for bottom left plot of group (a 'group' is an analysis of identical markers).

☒ All plots are contour plots with outliers or pseudocolor plots.

☒ A numerical value for number of cells or percentage (with statistics) is provided.

Methodology

Sample preparation	Human CD34+ HSPCs were treated with DAPI (Miltenyi Biotec), Live/Dead Fixable Violet dye (ThermoFisher), or DyLight 800 Maleimide (Thermo Scientific) in FACS buffer for 5-20 min to discriminate live and dead cells. If treated with antibodies, cells were washed with cell staining buffer (CSB) and resuspended with antibodies diluted in CSB for 30 min. Cells were washed with CSB and resuspended in FACS buffer.
Instrument	CytoFLEX (Beckman Dickinson) or FACS Aria sorter (Beckman Dickinson)
Software	Data collection was done using FACS Diva software and data analysis was done using FlowJo 10 software.
Cell population abundance	Any sorted samples were >95% pure as determined by post-sort flow cytometry purity analysis
Gating strategy	For samples analyzed on the CytoFLEX: FSC-H/SSC-H was used to gate for starting cell population. Singlets were selected with FSC-H/FSC-A plots. Gating was done to determine positive/negative populations based on negative control cells for each marker. For samples sorted on Aria: FSC-A/SSC-A was used to gate for starting cell population. Singlets were selected with SSC-H/SSC-A and FSC-H/FSC-A plots. Gating was done to determine positive/negative populations based on negative control cells for each marker.

☒ Tick this box to confirm that a figure exemplifying the gating strategy is provided in the Supplementary Information.
